# Optimal design and manufacture of variable stiffness laminated continuous fiber reinforced composites

**DOI:** 10.1038/s41598-020-73333-4

**Published:** 2020-10-05

**Authors:** Narasimha Boddeti, Yunlong Tang, Kurt Maute, David W. Rosen, Martin L. Dunn

**Affiliations:** 1 Digital Manufacturing and Design Centre, Singapore University of Technology and Design, Singapore, Singapore; 2 School of Mechanical and Materials Engineering, Washington State University, Pullman, USA; 3 Ann and H. J. Smead Aerospace Engineering, University of Colorado, Boulder, USA; 4 George W. Woodruff School of Mechanical Engineering, Georgia Institute of Technology, Atlanta, USA; 5 College of Engineering, Design and Computing, University of Colorado, Denver, USA

**Keywords:** Mechanical engineering, Structural materials

## Abstract

Advanced manufacturing methods like multi-material additive manufacturing are enabling realization of multiscale materials with intricate spatially varying microstructures and thus, material properties. This blurs the boundary between material and structure, paving the way to lighter, stiffer, and stronger structures. Taking advantage of these tunable multiscale materials warrants development of novel design methods that effectively marry the concepts of material and structure. We propose such a design to manufacture workflow and demonstrate it with laminated continuous fiber-reinforced composites that possess variable stiffness enabled by spatially varying microstructure. This contrasts with traditional fiber-reinforced composites which typically have a fixed, homogenous microstructure and thus constant stiffness. The proposed workflow includes three steps: (1) *Design automation*—efficient synthesis of an optimized multiscale design with microstructure homogenization enabling efficiency, (2) *Material compilation*—interpretation of the homogenized design lacking specificity in microstructural detail to a manufacturable structure, and (3) *Digital manufacturing*—automated manufacture of the *compiled* structure. We adapted multiscale topology optimization, a mesh parametrization-based algorithm and voxel-based multimaterial jetting for these three steps, respectively. We demonstrated that our workflow can be applied to arbitrary 2D or 3D surfaces. We validated the complete workflow with experiments on two simple planar structures; the results agree reasonably well with simulations.

## Introduction

Advanced manufacturing methods such as additive manufacturing (AM, or 3D printing), robotic fabrication, and 3D knitting allow manipulation of matter in new and interesting ways, thereby enabling innovative fabrication approaches to both improve existing products and create novel products^1,2^. These methods are enabling design and manufacture of structures with complicated geometries while also allowing synthesis of advanced multiscale materials with intricate microstructures^3–12^. These multiscale materials with complex geometrical arrangement of the constituents allow emergence of interesting material properties and overall behavior. This is empowering engineers to design *architected materials*^13^, examples of which include lattices and composites. Architected materials push the envelope of material property space (e.g., density, stiffness, and strength in the context of structural mechanics) beyond natural limits. This ability to tailor the material response at the microscale with architected materials unlocks extensive design freedom. In addition, advanced manufacturing allows the realization of spatially varying microstructures and thereby enables design of structures with complex material property gradients. Such material gradients are commonly found in biological materials such as wood, bamboo, bone, teeth, nacre, insect, and lobster cuticle. These materials are known to possess excellent mechanical stiffness, strength and toughness while being lightweight stemming from the synergetic arrangement of the constituent materials^14^. Advanced manufacturing, thus, gives designers the ability to simultaneously build structures and tailor material properties. This essentially blurs the distinction between material and structure. Traditional design practices, however, decouple material choice from structural design and thus are ill-equipped to handle the extensive design freedom that architected materials and advanced manufacturing provide. Novel design methods and workflows that take advantage of this extensive design freedom are needed.

Topology optimization (TO), which deals with optimal arrangement of materials given a geometrical design domain and design constraints, has recently emerged as a widely used advanced design methodology^15,16^. Mathematically, TO can be formulated as a partial differential equation (PDE) constrained optimization problem with the design variables being the physical parameters that appear in the PDE. TO can incorporate large a number of design variables, thus making it an ideal complement for advanced manufacturing methods with high degree of design freedom such as multimaterial AM^17^. This also enables the concept of multiscale TO where TO is adapted to multiscale design problems with the goal of simultaneously optimizing both the microstructure and macroscale topology. Several design approaches based on multiscale TO^9,18–44^ have been proposed. Most of these approaches focus on two-scale TO. Layered microstructures with multiple length scales known as *rank-n laminates* (*n* refers to number of hierarchical layers) have been mathematically proven to be optimally stiff^18,29,33,45^. However, these microstructures require scale separation across more than two length scales and hence, are not readily manufacturable. From here on, we take multiscale TO to refer to two-scale TO approaches that do not consider rank-n laminates.

Multiscale TO approaches can be broadly categorized into two, based on microscale optimization methodology. The first set of approaches assumes spatially varying microstructural topology and utilize numerical homogenization to bridge the scales^22,39,43,44^. This involves solving a microstructural optimization problem at each material point along with a macroscale optimization problem leading to large computational costs. Additionally, connectivity between varying microstructural topologies needs to be accounted for during optimization, otherwise the optimized structures are not manufacturable. The second set of approaches uses either a fixed microstructural topology^20,21,24,25,27,28,30,31,34–37,41,42,46^ or a set of pre-computed microstructural designs^9,19,32^ which reduces the computational cost considerably albeit at the expense of design freedom. The connectivity is ensured either through the inherent choice of the microstructure (e.g., two-phase microstructures^30,31,47^) or through post-optimization processing^24,27,33,34,37,41^.

We formulated a general design to manufacture workflow for additive manufacturing in our earlier work^30,31,47^ that falls into the second category of multiscale TO methods. This workflow consists of three steps: (1) *design automation*, (2) *material compilation* and (3) *digital manufacturing*. Given a design problem characterized by a prescribed design domain, objectives and constraints, design automation constitutes efficient synthesis of an optimized multiscale design. Computational efficiency is enabled by a specified parameterized microstructural topology and numerical or analytical homogenization of the microstructure. Homogenization involves representation of the inhomogeneous microstructure as a homogenous medium through an effective material stiffness tensor that depends on the microstructural geometrical descriptors such as orientation and volume fraction of fibers. This, however, renders the optimized designs devoid of geometrical detail (i.e., exact arrangement and connectivity of the constituents) at the microscale. Material compilation, then, connects the optimized design to a physically realizable structure through efficient computational geometry methods. Digital manufacturing, finally, deals with manufacture of the optimized design via computer integrated manufacturing systems.

In our previous work, we have adapted and demonstrated this workflow for short-fiber composites^30,31^ as well as functionally graded digital materials^47^. In this paper, we present the extension of our workflow to design optimization of continuous fiber composite structures with spatially varying fiber microstructure. Fiber reinforced composites (FRCs) are typically made of high strength fibers (e.g., carbon fibers, glass fibers, Kevlar, boron) embedded in a polymer matrix (e.g., epoxy, PEEK, PEI). FRCs possess several attractive properties when compared to metals such as high specific stiffness and strength, low coefficient of thermal expansion, corrosion resistance and high fatigue strength^48^. Thus, FRCs find extensive use in aerospace, automotive, marine, energy, space, construction and sporting goods industries^48^. Modern aircrafts such as Boeing 787 and Airbus A350 XWB feature FRC structures prominently^49–51^. FRCs also play a key role in the current and future development of efficient electric aircrafts, unmanned aerial vehicles, and electric cars, among others.

FRC structures are commonly designed and manufactured in a laminate form where each lamina or layer consists of aligned fibers. The alignment of fibers allows construction of structures tailored to a specific loading condition (e.g., uniaxial, biaxial). However, the fiber orientations in each layer are typically fixed, i.e., the composite microstructure is spatially uniform and exhibits *constant* stiffness. This may result in sub-optimal fiber orientations for structures with complex geometries and/or loading conditions. To overcome this shortcoming, laminates with spatially varying fiber orientations have been proposed and are referred to as variable stiffness laminates (VSLs)^52^. In a VSL, typically, fiber orientation is varied continuously within each layer to obtain variable stiffness at the microscale. In other words, the fibers follow simple straight lines in constant stiffness laminates (CSL) while following a curvilinear path in VSLs. VSLs can be fabricated with prepregs via advanced robotic fiber placement techniques such as automated fiber placement (AFP) and automated tape layup (ATL)^12,51^. Tailored fiber placement (TFP) based on embroidery with rovings also enables VSL fabrication^53^. Additive manufacture of VSLs is also gaining traction. Examples include methods based on material extrusion that involve extrusion of thermoplastic polymer coated continuous fiber filaments^54,55^ and robotic 3D printing known as CF3D that impregnates continuous fibers with a thermoset polymer resin in-situ and rapidly cures the extruded fiber-resin combination^56^.

Extensive work has been done on design optimization of VSLs^57–59^. Most of the earlier efforts focus on the microstructural optimization i.e., fiber path (or fiber volume fraction in some cases^60,61^) and do not deal with the macroscale structure. Recent efforts have started utilizing topology optimization to couple microstructure optimization with macroscale material layout design^20,21,40,62–65^. The fiber path to be optimized is usually parametrized by use of discrete nodal or elemental fiber angles directly^61,66–68^, by a family of curves (the curves were used to define either the fiber orientations or paths e.g., linear curves, Bezier curves, Lagrange polynomials)^69–75^, lamination parameters^76–82^ and polar lamination parameters^83^. When using a family of curves, a manufacturable fiber layout is readily available, but this restricts the design space. Use of lamination parameters over fiber angles leads to convex feasible regions available for optimization, but requires further computational processing to determine the fiber angles^59^. When fiber angles are available (either directly from optimization or through lamination parameters), a post-processing step is utilized to determine the fiber paths or layout. One commonly used approach is the streamline methodology where fiber paths are obtained from the streamlines of the optimized fiber angle field^65,67,79,81,82,84^. However, this does not guarantee uniform fiber distribution and thus could lead to unwanted overlaps or gaps when AFP/ATL techniques are used to realize the optimized structures. Another common approach with level set based topology optimization is to use the parallel offsets of the contours of the optimized structure to orient the fibers^22,65^. This, however, reduces the design freedom by requiring the fibers to orient along the contours of the structure and may lead to sub-optimal results.

For the design automation step of our workflow, in this work and as in our previous studies^30,31^, we adapted topology optimization with the Mori–Tanaka homogenization scheme^85–88^ to simulate the homogenized material response of FRCs. The Mori–Tanaka scheme is an analytical homogenization method which predicts the local effective material stiffness given the local fiber and matrix moduli, fiber orientation, fiber aspect ratio and fiber volume fraction. Use of an analytical homogenization scheme reduces computational complexity and allows us to consider spatial variation of not just fiber orientations, as is typically done in VSL design, but also fiber volume fraction. We used the same digital manufacturing approach as in our previous studies, viz., voxel-based multimaterial jetting to realize the optimized composites structures. However, the simple computational geometry technique from our previous studies for short fibers^30,31^ is not applicable to continuous fibers. A new material compilation algorithm was developed based on the *stripe patterns* algorithm proposed by Knöppel et al.^89^.

The *stripe patterns* algorithm is a geometry processing algorithm that can generate stripes on a 3D surface with desired spacing and aligned along an input vector field, while generating singularities where it cannot do so. In our context, the input vector field is defined by the optimized fiber orientations while the fiber volume fractions define the spacing. The stripes, thus, realize the spatially varying fiber-based microstructure. A similar algorithm proposed by Pantz and Trabelsi^41^ deals with determining a scalar field (akin to a stream function), the gradient of which is aligned along a given vector field. The isolines of the scalar field are then used to *project* the chosen microstructure (fibers, rectangular lattices etc.). It is to be noted that no consideration to the spacing is given in Pantz and Trabelsi’s algorithm which is a prominent feature of the stripe patterns algorithm. Variations of Pantz and Trabelsi’s algorithm have been adapted by others to realize fiber-based or lattice-based spatially varying microstructures in 2D and 3D structures^24,27,33,36,37,41^. Wu et al.^34^, used a graph-based field-aligned meshing algorithm to achieve the same. The stripe patterns algorithm on the other hand is capable of handling vector fields on any arbitrary surface (i.e., 2D and 2.5D) and thus making it an ideal choice for FRCs which are typically layered.

We demonstrate here our multiscale TO approach with design of maximally stiff FRC laminates under various commonly encountered boundary and loading conditions. This is one of the first approaches to tackle 2.5D (i.e., layered) structures and complements the existing 2D and 3D multiscale TO approaches. We also connect our design approach to an additive manufacturing method through the stripe patterns algorithm. We demonstrate the unique ability of our workflow to design and fabricate continuous FRCs with spatially varying fiber orientations as well as volume fractions. To demonstrate our complete design to manufacture workflow and experimentally validate the approach, we 3D printed two simple planar structures with a rigid polymer (~ 1 GPa) and a soft elastomer (~ 1 MPa) for the fibrous and matrix materials respectively. The details of the workflow, results of the laminate design optimization problems, and the experiments follow.

## Results

### Design and manufacturing workflow

**Figure 1:**
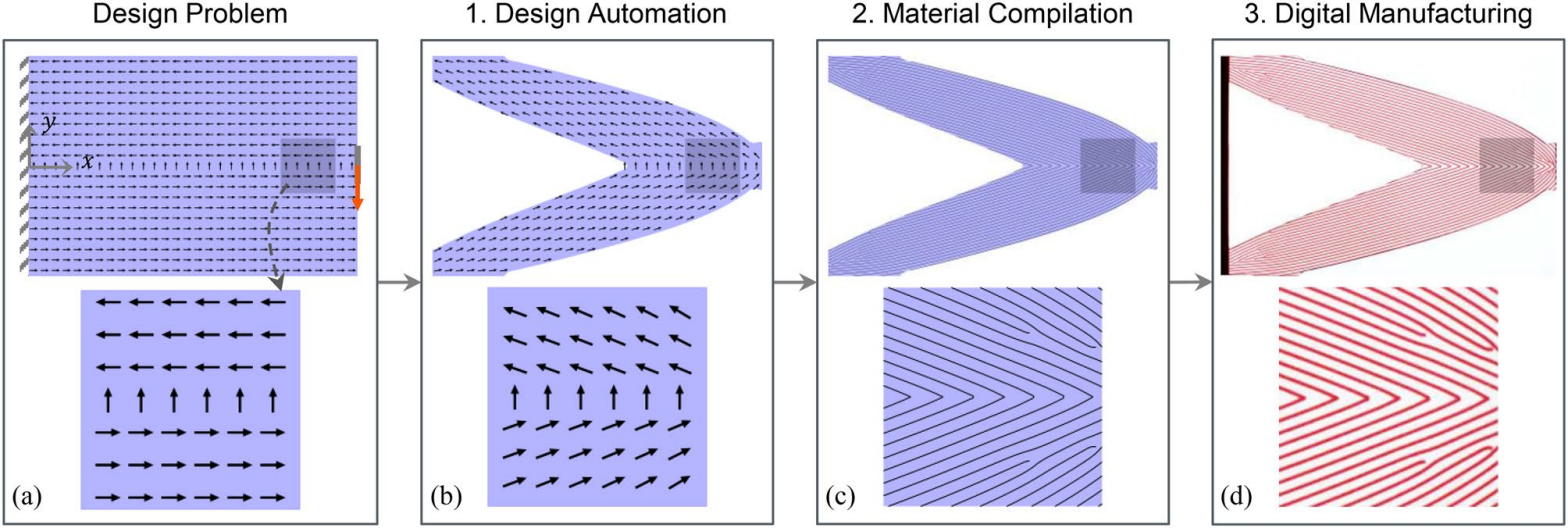
Simultaneous multiscale design optimization to manufacture workflow adapted for variable stiffness continuous fiber composites: (**a**) Design problem formulation showing the design domain, load and boundary conditions for an optimally stiff short beam. Also shown are the initial fiber orientations with the help of arrows, (**b**) Design automation—results from topology optimization showing the optimized macrostructure and microstructure (just fiber orientations in this case), (**c**) Material compilation—realization of a manufacturable fiber layout (depicted as black curves) obtained from stripe patterns algorithm, and (**d**) Digital manufacturing—3D-printed structure via voxel-based multi-material jetting for illustrative purposes with clear and magenta colored materials for the matrix and fiber respectively. Insets show a zoom-in of the dark shaded region with either fiber orientations (in **a**,**b**) or fiber layout (**c**,**d**).

Figure 1 presents our design and manufacturing workflow adapted for simultaneous multiscale design optimization of continuous fiber composites with spatially varying microstructure. The workflow starts with design problem specification followed by the three key steps of: (1) *design automation*, (2) *material compilation* and (3) *digital manufacturing*. The various steps of the workflow are illustrated using the standard problem of an optimally stiff 2D short beam. These steps are discussed in detail in the following sub-sections.

### Design problem

In the context of structural composites, the design problem formulation involves aggregation of design domain (i.e., physical extents the design can span), loading conditions, boundary conditions, matrix and fiber material properties, design objectives, and constraints. Typical objectives for design of composites include weight, stiffness, structural failure (buckling), material failure, Eigen frequencies etc. We focused exclusively on stiffness in this work, but the framework can easily be extended to other structural performance metrics. Design constraints typically arise from cost considerations (material usage i.e., volume or weight) or manufacturing (e.g., physical limits on obtainable fiber curvature).

The design domain for the 2D short beam in Fig. 1a is a rectangle of length 120 mm and width 80 mm. A nominal thickness of 20 mm was assigned to the linear quadrilateral finite elements used. The structure was assumed to be rigidly clamped on the left edge and designed to support a bending load applied through a vertical displacement of 1 mm as highlighted by the red arrow in Fig. 1a. The matrix and fiber materials were assumed to be linear elastic and possess Young’s moduli of 1.22 MPa and 1.03 GPa respectively, values obtained from our previous study^31^. The Poisson’s ratio was assumed to be 0.4 for both.

### Design automation

Design automation entails automated synthesis of an optimized structure given a desired design objective and cost/manufacturing constraints expressed mathematically. To achieve this, we adapted topology optimization (TO). TO couples finite element (FE) analysis with a gradient-based optimization algorithm to iteratively update and evolve the structural design from an initial guess to a final design that optimizes the given design objective and satisfies specified constraints (see our earlier work^30,31^ for a detailed workflow). During each design iteration, the design objective and constraints are calculated based on a finite element simulation and their gradients are evaluated with respect to the chosen set of optimization variables, $s$. The gradients are then used by the optimization algorithm to update the design and the iterations are stopped when the design converges. Mathematically, a general TO problem is stated thus:

1 $$\begin{array}{lll}\underset{s}{min} & z\left({\varvec{u}}\left(s\right),s\right) & \\ s.t.& {g}_{i}\left({\varvec{u}}\left(s\right),s\right)\le 0& \quad i=\mathrm{1,2},\cdots ,m\\ & {h}_{j}\left({\varvec{u}}\left(s\right),s\right)=0& \quad j=\mathrm{1,2},\cdots ,n\\ & {s}_{l}\le s\le {s}_{u}& \end{array}$$

here, $z$ is the objective, ${g}_{i}$ and ${h}_{j}$ are the $i$th inequality and $j$th equality constraints, respectively. The objective and constraints are all functions of the optimization variables, $s$. Some of these functions show explicit dependence on $s$ (e.g., mass, volume) while others show implicit dependence through the state of the structure described by structural displacements, ${\varvec{u}}$ (e.g., stiffness, maximum stress). The displacements are dependent on structural topology and material property distribution (e.g., Young’s modulus, mass, thermal conductivity), which are in turn defined as functions of the optimization variables. The optimization variables are typically defined as FE mesh elemental or nodal variables and hence $s=s({\varvec{x}})$, ${\varvec{x}}(x,y,z)$ being the position vector.

The displacements due to deformation of the structure subjected to prescribed loads and boundary conditions are obtained from the standard FE analysis which solves the elastostatic equilibrium equation: $\nabla {\varvec{\upsigma}}\left({\varvec{u}}(s)\right)+{\varvec{b}}=0$, where ${\varvec{b}}$ is the body force vector and ${\varvec{\upsigma}}$ is the 2nd order stress tensor. The stress tensor is given by the generalized Hooke’s law, ${\varvec{\upsigma}}=\mathbf{C}\left(s\right){\varvec{\upepsilon}}\left({\varvec{u}}\left(s\right)\right)$ where $\mathbf{C}$ is the 4th order material stiffness tensor and ${\varvec{\upepsilon}}=\left(\nabla {\varvec{u}}+\nabla {{\varvec{u}}}^{T}\right)/2$ is the 2nd order tensor for small strains.

We used a density based TO approach where a normalized fictitious density, $\rho (s)$, represents the macroscale topology (i.e., material layout). Each material point is attributed a density value such that $0<{\rho }_{min}\le \rho \left(s\right)\le 1$. A non-zero value is used for the lower bound of $\rho$ (i.e., ${\rho }_{min}$) to avoid ill-conditioning of the FE problem. This density field, typically, is used to vary the isotropic material properties, such as Young’s modulus or thermal conductivity, between a lower and upper bound. Since FRCs are not isotropic, we varied the 4th order material stiffness tensor, $\mathbf{C}\left(s\right)$ such that:

2 $$\mathbf{C}\left(s\right)={\mathbf{C}}_{1}+\rho {\left(s\right)}^{p}({\mathbf{C}}_{2}(s)-{\mathbf{C}}_{1})$$

here, ${\mathbf{C}}_{1}$ and ${\mathbf{C}}_{2}$ are the material stiffness tensors of two different material compositions (or void when ${\mathbf{C}}_{1}$ is a null tensor) while the exponent $p$ is a constant. When $\rho =$ 0, it implies presence of material with a constant stiffness tensor ${\mathbf{C}}_{1}$ whereas $\rho =1$ implies presence of composite with spatially varying stiffness ${\mathbf{C}}_{2}(s)$. When needed, intermediate values are penalized by setting $p=$ 3 following SIMP (solid isotropic material with penalization)^15^ and PAMP (porous anisotropic material with penalization)^42^ approaches. We used the standard linear filter^90^ and projection filter^91^ as necessary to regularize the optimization problem (see Section 1.2, SI).

To enable efficient modeling of the multiscale nature of the FRCs, we coupled FE analysis with analytical homogenization approach where the effect of the microscale mechanics on the macroscale response of the composite are accounted for in an averaged sense. We used the well-known Mori–Tanaka homogenization method based on Eshelby’s inclusion problem. This method treats fibers as prolate spheroids to arrive at an effective macroscale material stiffness tensor and identifies the local fiber orientation ($\theta$), fiber volume fraction ($f$), and fiber aspect ratio ($\alpha$) as the microstructural parameters in addition to the matrix and fiber isotropic material properties, viz., Young’s moduli (${E}_{m}$ and ${E}_{f}$) and Poisson’s ratio (${\nu }_{m}$ and ${\nu }_{f}$). We can consider any combination of these microstructural parameters (including matrix and fiber moduli) as design variables with our framework but limited ourselves to $f$ and $\theta$ for the sake of simplicity. We assumed $\alpha \gg$ 1 i.e., long, continuous fibers. Thus:

3 $${{\varvec{C}}}^{{\varvec{H}}}(s)={{\varvec{C}}}^{{\varvec{H}}}\left({E}_{m},{E}_{f},{\nu }_{m},{\nu }_{f},\alpha , f(s),\theta (s)\right)$$

It should be noted that we need only one angle to describe the orientation of a fiber in this study as opposed to two angles we used in our previous study with 3D composite design^30^. This is because the fiber is implicitly assumed to always lie on the surface of the laminate, thus making $\theta$ the fiber orientation on the local tangent plane with respect to a chosen coordinate system at any given point. Fiber orientations could either be directly used as design variables (known as continuous fiber angle optimization or CFAO) or parametrized following methods such as isoparametric projection method^20^, normal distribution fiber optimization^92^, and discrete–continuous parametrization^93^. We chose to use the straightforward CFAO approach to keep things simple given the focus here is to demonstrate the design to manufacturing workflow.

### Material compilation

By design and due to the use of homogenization, the optimized microstructural parameters obtained from the design automation step do not readily present a complete geometrical description of the microstructure. Thus, we conceptualized a material compilation step which, with the help of a suitable computational geometry technique, *compiles* a physically realizable local material microstructure given the abstract optimized microstructural parameters. This compilation algorithm needs to be tailored for the microstructure in use and, for continuous fiber composites, we adapted Knoppel et al.’s^89^ stripe patterns algorithm which generates a pattern of stripes that could be likened to fibers. The algorithm aligns stripes along an input vector field, $X$ on any given manifold with control over stripe spacing. This is achieved by solving for a periodic scalar function, $\xi$ defined over the whole manifold and whose gradient is aligned with ${X}_{\perp }$ (a vector field perpendicular to the input field $X$, $X\cdot {X}_{\perp }=0$ and lying on the manifold). Mathematically, $\nabla \xi =\nu {X}_{\perp }$ where $\nu$ is the stripe frequency which we linked to the fiber spacing and thus volume fraction (see Section 2, SI). The scalar function, $\xi$ could be defined as the argument of a complex function, $\varphi$ with unit norm ($\left|\varphi \right|=$ 1), thus guaranteeing periodicity. This means $\nabla \varphi =i\nu {X}_{\perp }\varphi$ and Knoppel et al. note that solving this leads to the trivial solution of $\varphi =$ 0 for non-integrable vector fields (i.e., non-curl free) as desired spacing and alignment cannot be achieved in regions with high curl. They work around this by relaxing the unit norm condition on $\varphi$ and instead solve for a complex function, $\psi =a\varphi$ which preserves the periodicity but rescales $\varphi$ by $a\ge 0$ ($a$ is a real valued function). Imposing the condition $\Vert \psi \Vert =$ 1, i.e., unit L^2^-norm and solving $\nabla \psi =i\nu {X}_{\perp }\psi$ for $\psi$ gives the best approximation for $\varphi$ and thus $\xi$. Mathematically, this is equivalent to solving for the minimum eigenvalue and the corresponding eigenfunction of a generalized eigenvalue problem. The algorithm thus accommodates alignment in the non-integrable regions by creating *singularities* where $\psi$ is scaled down to zero (see Fig. S10, SI).

### Digital manufacturing

We used Stratasys Objet500, a voxel-based multimaterial jetting 3D printer, to fabricate the optimized composite structures. This 3D printing technique uses print heads equipped with ultraviolet (UV) lamps, a roller, and multiple nozzles. The nozzles jet out liquid droplets of a desired UV curable polymer resin drawn from material reservoirs and deposit at specified locations onto a planar print bed surface following a computer-generated deposition pattern. The UV lamp and rollers on either side of the nozzles cure and flatten the deposited droplets, respectively. This flattened droplet forms the basic building block, termed a voxel (volume element). The whole process is precisely engineered to realize voxels of dimensions 42.3 × 84.7 × 30 µm. This is to say that the printer has a resolution of 42.3 µm (600 dpi) along the direction in which the print head moves while it is 84.7 µm (300 dpi) along a direction perpendicular to the print head movement within the plane of the print bed. The printer is capable of jetting out three different resins in addition to a support material that can be dissolved after printing. The printing process starts with a 3D model that is sliced into layers; each one voxel thick (30 µm). For each layer, three black and white bitmaps are generated for the three available materials. In each bitmap, the white colored pixels encode the voxel locations of the material associated with the bitmap. The resolution of the bitmaps and the number of slices/layers needed are determined from the extents of the bounding box of the structure to be printed.

It is impractical to represent the FRCs using traditional 3D modeling software due to the large number of surfaces present. We circumvented this problem by converting the results from design automation and material compilation directly to bitmaps without building any 3D models. We developed a simple voxelization routine based on open-source software OpenVDB^94^ and VTK (Visualization Toolkit)^95^. OpenVDB provides a hierarchical data structure that enables efficient representation of voxel grids and a suite of associated tools to manipulate the data structures. OpenVDB thus enables us to deal efficiently with voxel-based structural representations which have a voxel density of 9.3 million per cubic centimeter for the Stratasys Objet500 and a few billion voxels for even a relatively small structure.

Our routine takes as input the surface representation of the optimized laminate topology, polylines representing the fibers and fiber radius. We used VTK to read the input files and OpenVDB to generate an efficient voxel representation of the matrix and the embedded fibers (details in Section 2.1, SI). The required sets of bitmaps are then generated from OpenVDB’s voxel representation. We also used our routine to impart color to the fibers for better visualization. This is done by randomly assigning the fiber voxels to VeroMagenta (a magenta colored rigid polymer) with a probability of 10% while the rest are Vero Clear (a transparent rigid polymer with properties nearly identical to VeroMagenta).

### Design optimization of laminated fiber reinforced structures

We optimized laminated composite structures with Euclidean as well as non-Euclidean geometries to demonstrate the capabilities of our workflow. The Euclidean geometries that we optimized are planar or cylindrical surfaces (Gaussian curvature = 0) while non-Euclidean geometries are a sphere (Gaussian curvature > 0) and a hyperbolic paraboloid (Gaussian curvature < 0). A discussion on the optimization problem setup and the optimized designs with the fiber layouts obtained from the stripe patterns algorithm for each case follows.

### Planar structures

We optimized two simple planar composite structures that find use in several applications. The first one is a plate with a hole under tensile loading and plane stress conditions, while the other is a laminated plate with a localized bending load. Owing to their simplicity, we used the planar optimized structures to fabricate and experimentally validate the workflow. So, we used the experimentally obtained equivalent Young’s moduli of TangoPlus (${E}_{m}=$ 0.97 MPa) and VeroMagenta/VeroClear mix (${E}_{f}=$ 0.85 GPa) for the matrix and fiber respectively in these optimization problems (see Section 3.1, SI). The Poisson’s ratios were assumed to be 0.4 for both. We set ${\mathbf{C}}_{1}=0$, i.e., void in Eq. (2) for both optimization problems.

### Plate with a hole

**Figure 2:**
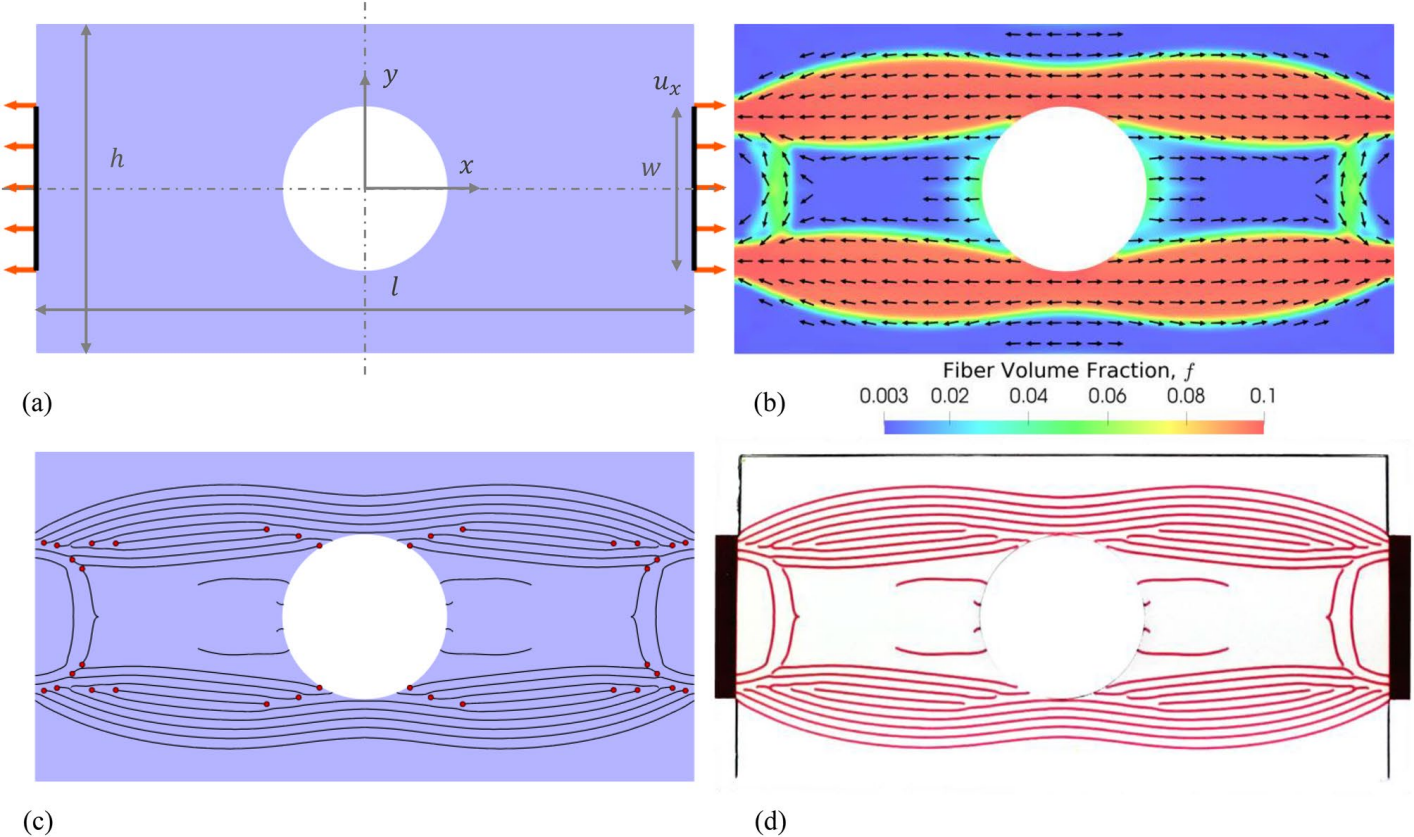
Design optimization of a composite plate structure with a hole: (**a**) Problem setup with the design domain, tension load (red arrows), symmetry planes (dashed lines) and geometrical parameters identified ($l =$ 80 mm, $h =$ 40 mm, and $w =$ 20 mm). (**b**) Optimized design from the design automation step: The color map indicates the spatially varying fiber volume fraction, $f$, with blue representing $f \approx$ 0 and red $f =$ 10% and the arrows indicate the optimized fiber orientations. (**c**) Results of the material compilation algorithm with the black curves representing individual fibers and red dots refer to singularities created by the stripe patterns algorithm to improve alignment in high curl regions, (**d**) 3D printed sample for visualization with a single layer of fibers using VeroMagenta (fibers) and VeroClear (matrix). End tabs used for loading the actual structure are also seen.

A plate with a hole is a well-studied and frequently used composite structure. The hole in the plate leads to reduced stiffness and strength under tension or compression, thus necessitating careful design of the fiber composite. Generally, the fiber orientations are optimized to obtain a conventional or curvilinear fiber layout^66^. Here, we optimized for both fiber orientations, $\theta$ ($-\frac{\pi }{2}\le \theta <\frac{\pi }{2}$) as well as the fiber volume fractions, $f$ ($0\le f\le 0.1$) while keeping the macroscale topology fixed (i.e., $\rho ({\varvec{x}})=1$). The maximum fiber volume fraction is limited to 10% due to the limited printer resolution. The overall fibrous material usage, ${V}_{f}$, was constrained to be at most 5% of the design domain volume, ${V}_{\Omega }$, i.e., ${V}_{f}\le$ 0.05 ${V}_{\Omega }$. The homogenized composite material stiffness through the thickness was assumed to be constant and hence a 2D model with plane stress state was used. The problem setup is shown in Fig. 2a. The design domain has lateral dimensions 80 × 40 mm and a nominal thickness of 8 mm. The tension load was applied as a uniform horizontal displacement of the highlighted boundary regions and indicated by the red arrows. We took advantage of the symmetry of the problem and simulated only the right, top quarter. An isotropic linear filter with a smoothing radius, ${r}_{s}=$ 1 mm was applied to the optimization variables, $s$, so that the resulting $f$ and $\theta$ vary smoothly.

The result from the design optimization step is shown in Fig. 2b where the colors indicate spatially varying fiber volume fraction, $f$, with blue implying pure matrix (i.e., $f=0$) and red denoting a composite with 10% fiber volume fraction. Intermediate colors indicate a composite with $0<f<0.1$. The spatially varying $\theta$ are depicted with oriented arrows at sampled locations. It is to be noted that $\theta$ and hence the arrows can be flipped 180° with no effect on the mechanics, but a coherent direction is needed to define the vector field that forms the input to the stripe patterns algorithm. Figure 2c shows the result of the stripe patterns algorithm where black curves depict the optimized fiber layout and red dots the singularities created by the algorithm. The optimized design orients the fibers essentially along the principal stress lines to stiffen the structure. The highest stress occurs along the hole periphery on the *y*-axis, as expected. Figure 2d shows a layer of the composite that was 3D-printed with VeroMagenta and VeroClear for visualization of the fiber layout (see Section 3.2, SI for actual samples).

### Laminated plate

**Figure 3:**
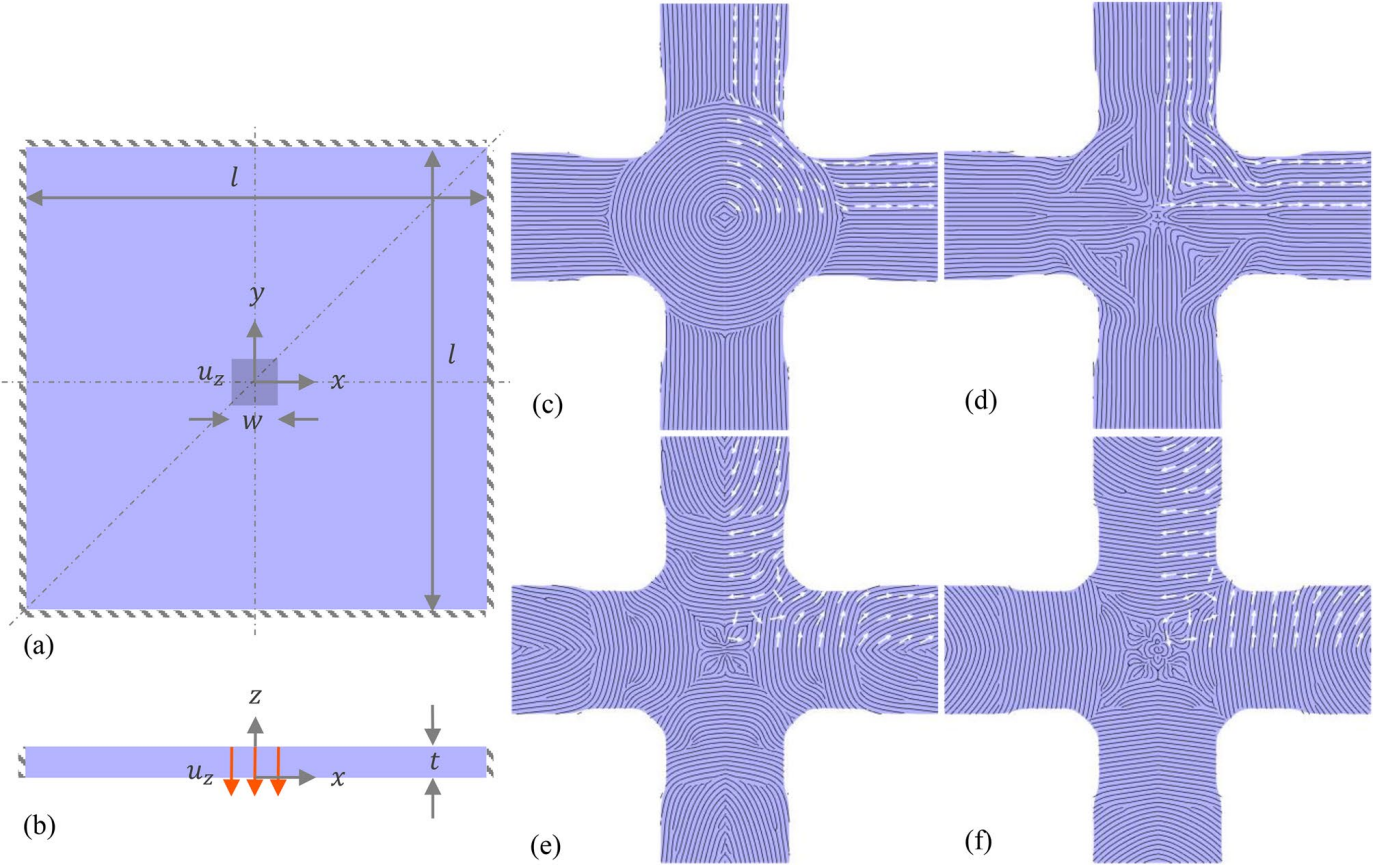
Laminated composite plate optimization problem setup and results: (**a**) Top and (**b**) side views of the design domain with the bending load (shaded region with dimensions $w \times w$ in the middle) and geometrical parameters identified ($l =$ 100 mm, $t =$ 7.68 mm, and $w=$ 10 mm). The symmetry planes are also identified in the top view. (**c**–**f**) Optimized topology (macrostructure in light blue) and fiber orientations (microstructure, depicted by white arrows) for layers 1 to 4 (stacking sequence is **c**–**f**) of 8 total layers. The physically realizable fiber layout from the stripe patterns algorithm is also shown in black for each layer.

A multiscale problem was formulated to design an optimally stiff square laminated plate with 8 layers (an arbitrary choice) to simultaneously optimize the macroscale topology and microstructure. The overall material usage was constrained to be within 50% of the design domain volume (100 × 100 × 7.68 mm). The plate was assumed to be clamped on all four sides and subjected to a bending load concentrated in the middle of the plate as shown in Fig. 3a,b. The bending load was applied in the form of a uniform displacement of 1 mm along the *z*-axis. Owing to the symmetry of the boundary and loading conditions, only a quarter of the structure was used to simulate with symmetry boundary conditions. In addition, the design was assumed to be symmetric about the diagonal axis in the *xy*-plane. The fiber volume fractions $f$ were kept fixed at 10% ($f({\varvec{x}})=$ 0.1) while the fiber orientations, $\theta$ ($-\frac{\pi }{2}\le \theta \le \frac{\pi }{2}$), in each of the 8 layers can vary independently. Thus, the density distribution $\rho$ describes the macroscale topology and $\theta$ the microstructure. Intermediate $\rho$ values were penalized by setting the SIMP exponent, $p$ to be 3 in Eq. (2). Additionally, an isotropic linear filter (${r}_{s}=$ 8 mm) in conjunction with a projection filter was applied to the density optimization variables, ${s}_{\rho }$ (${s}_{\rho }\subset s$) so that an effective macroscale design with clear distinction between void ($\rho ={\rho }_{min}=$ 0.001) and material ($\rho =$ 1) can be obtained. Simultaneously, an anisotropic filter (see Section 1.2, SI) was applied to the orientation optimization variables, ${s}_{\theta }$ (${s}_{\theta }\subset s$) with an in-plane smoothing radius of ${r}_{s}^{p}=$ 4 mm and a smoothing radius of 1 mm along the normal direction, ${r}_{s}^{n}=$ 1 mm. This anisotropic filter allows smooth variation of $\theta$ in the plane of the lamina while decoupling $\theta$ across the lamina.

Figure 3c–f show the optimized macroscale topology and the microstructure (i.e., fiber orientations depicted with white arrows at sampled locations) for the bottom four layers. The fiber layout obtained from the stripe patterns algorithm is also seen. The fiber layout of the other four layers can be found in the SI (see Fig. S6) and are nearly symmetric with respect to the mid-plane of the structure. The optimized topology has four arms connected to a central circular region, similar to the result we obtained for short-fiber composite structures^31^. The thickness of the laminate obtained is uniform owing to the large linear filter smoothing radius. However, the thickness can be optimized by simply choosing an anisotropic filtering scheme like the one we used for $\theta$ (see Section 1.4, SI).

### Experiments

Table 1 presents the results of the experiments performed on the 3D-printed planar structures. The singularities generated by the stripe patterns algorithm to accommodate non-integrable input fiber orientations result in fiber layouts with discontinuities and are essentially defects in the composite (see Fig. 2c and Section 2.2, SI). These singularities leave the matrix unreinforced leading to low stiffness regions that were not accounted for in the simulations. When the defect density (number of defects per unit volume) is high, this affects the structural stiffness adversely and thus leads to inaccurate interpretation of the simulation results. We, however, hypothesized that for structures with sufficient scale separation (macrostructure length scale $\gg$ fiber diameter), the defect density should be small and hence, the defects have a negligible effect on the structural performance. We, thus, printed the plate with a hole structure in three different lateral sizes each with a different degree of scale separation by holding the fiber diameter and thickness constant (see Fig. S19, SI). The three sizes include structures laterally scaled by two and three times the original simulated dimensions of 8 × 4 × 0.8 cm. These were labeled cases A, B and C with A referring to the smallest structure and C the largest.

**Table 1:** Planar structures: comparison of experiments and simulations.

Structure	Dimensions (label) (cm $\times$ cm $\times$ cm)	Number of defects	Defect density (cm^−3^)	Effective stiffness (N/mm)		
				Simulation	Experiment	
					Disconnected	Connected
Plate with a circular hole	8 $\times$ 4 $\times$ 0.8 (A)	292	12.65	84.36	50.55	62.1
	16 $\times$ 8 $\times$ 0.8 (B)	692	7.49		69.10	70.6
	24 $\times$ 12 $\times$ 0.8 (C)	952	4.58		64.58	–
Laminated plate	10 $\times$ 10 $\times$ 0.77	938	24.43	4.87	3.42	3.52

At the same time, the discontinuities in the fiber layout can be avoided by simply connecting the fibers with a simple geometry processing algorithm (see Section 2.1, SI). We, thus, printed the two smaller sized structures (cases A and B) with connected fiber layouts. The resulting structures, with connected fibers or not, have the same effective theoretical stiffness (force exerted to displace the loading region by 1 mm). The experimentally measured stiffness values along with the defect densities are shown in Table 1 (first row). We also employed digital image correlation (DIC) to obtain displacement maps from the deformed structure with case B (see Fig. S21, SI).

We observed improved effective stiffness with decreased defect density with the defect density being inversely proportional to the structural size supporting our earlier hypothesis. Case A with disconnected layout shows the highest discrepancy of 40.0% compared to the simulated stiffness of 84.36 N/mm and this improves to 26.0% when the fibers are connected. We measured higher stiffness values of 69.1 N/mm and 64.58 N/mm for cases B and C, respectively. These measured values, however, agree well with the effective stiffness value of 70.8 N/mm from an FE analysis where defects were simulated by setting fiber volume fraction to zero near the defects (see Fig. S22 and Table S3, SI). The structures with connected fibers showed significant improvement for case A and little improvement for case B. We surmise that even as the connected fibers help avoid matrix-rich regions, they do not provide an effective load transfer pathway and thus are of help only with high incidence of defects. The reduced stiffness in case C compared to case B could be attributed to distortion of the printed structure caused by the release of strain in TangoPlus that was built up during printing^96^ (see case C in Fig. S19).

The results for the laminated plate structure are also presented in Table 1 where the discrepancy is about 30% and the connected fiber layout showed little improvement. The defect density is twice that of the laminated plate, case A, as we can see rapid 90° changes in fiber orientations in each lamina (see Fig. S17, SI).

### Non-planar structures

The non-planar structures that we optimized for stiffness include a: (1) cylindrical tube subjected to a twisting load, (2) truncated spherical shell under a localized bending load and (3) a hyperbolic paraboloid-shaped roof supporting a distributed bending load. In all these structures, we optimized the distribution of the fibrous material within a fixed macroscale topology. The total fibrous material usage, ${V}_{f}$ was constrained to be within 20% of the design domain volume, ${V}_{\Omega }$, i.e., ${V}_{f}\le 0.2{V}_{\Omega }$ and $-\frac{\pi }{2}\le \theta \le \frac{\pi }{2}$.

With each structure, we set up two identical optimization problems with one significant difference with respect to the variation of fiber volume fractions, $f$. In one case, continuous variation of $f$ between 0 to 0.4 was allowed. This was achieved by simply setting $\rho \left({\varvec{x}}\right)=1$. In the other case, $f$ was restricted to discrete values of either 0 or 0.4 through penalization of intermediate $f$ values and a projection filter. To do this, we set ${\mathbf{C}}_{1}={\mathbf{C}}_{\mathbf{m}}$ (${\mathbf{C}}_{\mathbf{m}}$ is the matrix material stiffness tensor) and $p=3$ in Eq. (2) in addition to coupling $\rho$ and $f$ via $\rho \left(x\right)=f(x)/{f}_{max}$ (${f}_{max}=$ 0.4).

Optimized designs with discrete values of $f$ are more amenable to manufacturing techniques that use a fiber tape (AFP/ATL) where the volume fraction is inherently fixed. In contrast, optimized designs with continuous $f$ variation are not suitable for AFP, ATL or similar methods but were found to be stiffer and thus more optimal than their counterparts with discrete $f$. These are, however, readily manufacturable with voxel-based printing as we demonstrated with the planar structures and could prove more attractive when fabricated with fibrous materials comparable to glass-fiber or carbon-fiber.

For these problems, we assumed that the composite comprises of commonly used materials, viz., epoxy (${E}_{m}=$ 3 GPa, ${\nu }_{m}=$ 0.33) and carbon-fiber (${E}_{f}=$ 200 GPa, ${\nu }_{f}=$ 0.25). Each composite structure was assumed to have two laminae, each with a distinct fiber layout. As with the laminated plate problem, we achieved this by employing an anisotropic smoothing filter. With these non-planar structures, however, the ellipsoidal envelope used for linear filtering needs to be aligned with the local tangent plane (see Section 1.2 and Fig. S1, SI).

### Cylindrical structure

**Figure 4:**
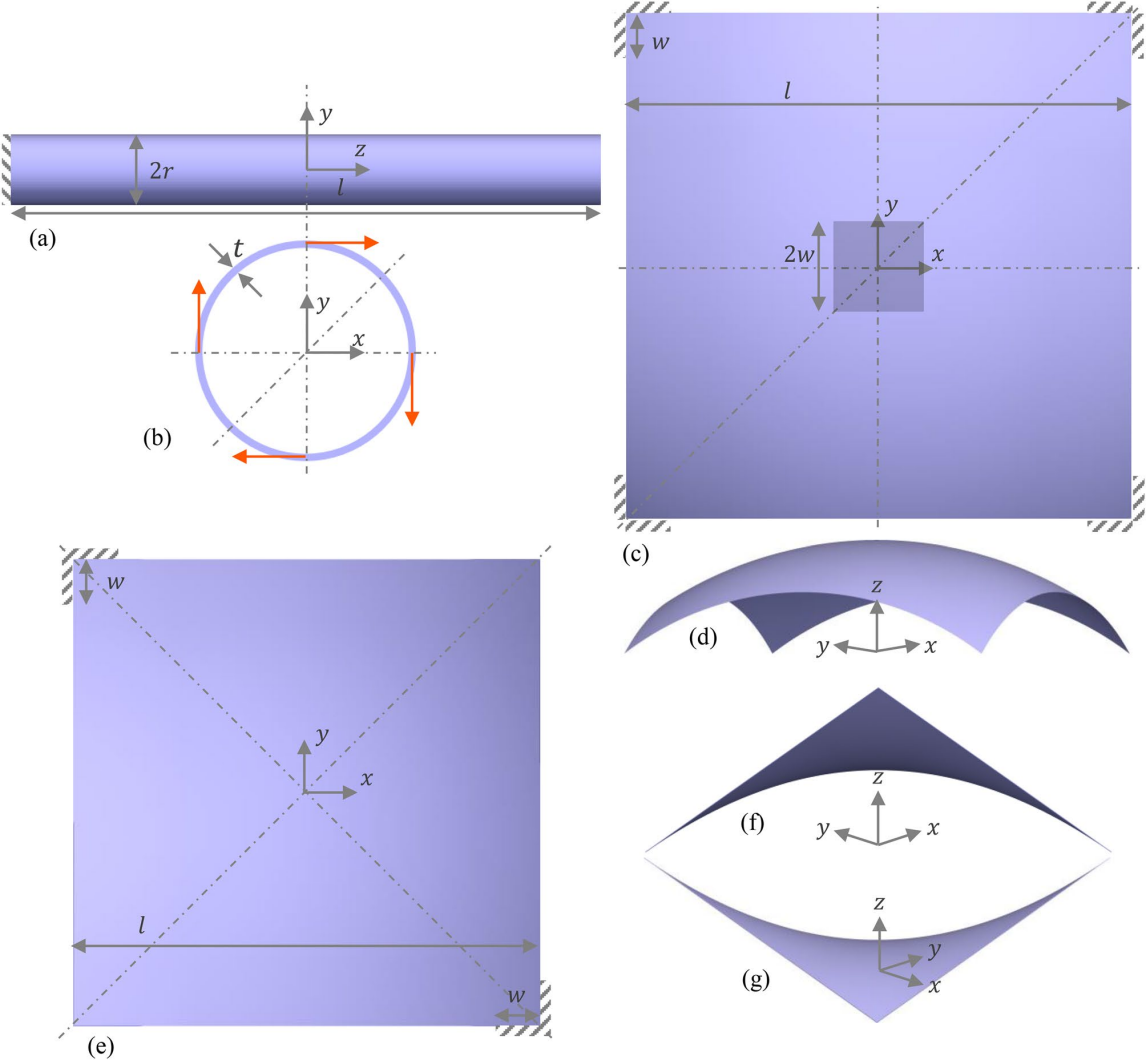
Non-planar structures optimization problem setups: (1) Cylindrical composite tube: (**a**) longitudinal view and (**b**) cross-sectional view of the design domain with boundary conditions, clockwise distributed twisting load (red arrows), design symmetry planes (dashed lines) and geometrical parameters ($l=$ 500 mm, $r=$ 30 mm, $t=$ 2 mm) identified, (2) Spherical-roof structure: (**c**) top view of the design domain with boundary conditions, bending load (shaded region of width $2w$ in the middle), design symmetry planes (dashed lines) and geometrical parameters ($l=$ 2 m, $w=$ 0.2 m) identified, (**d**) side view showing the curvature of the structure., (3) Hyperbolic paraboloid-roof structure: (**e**) top view of the design domain with boundary conditions, design symmetry planes (dashed lines) and geometrical parameters ($l=$ 10 m, $w=$ 0.1 m) identified, (**f**,**g**) are side views showing the curvature of the structure.

Composite tubes have extensive applications in lightweight frames for aerospace structures and sporting goods. They are also used as drive shafts for torque transfer. This motivated us to optimize cylindrical composite tubes subjected to clockwise twisting loads as shown in Fig. 4a,b. We chose dimensions of 500 mm for length, 60 mm for the tube diameter and 2 mm for the tube thickness that are representative of the commercially available tubes. The tube was assumed to be clamped on one end and a uniformly distributed traction load of 10 MPa was applied to the other end. The structure was assumed to possess eightfold symmetry in the *xy*-plane while being symmetric about the plane bisecting the structure along the *z*-axis. This necessitated constraints on the optimization variables as necessary (see Table S2, SI).

**Figure 5:**
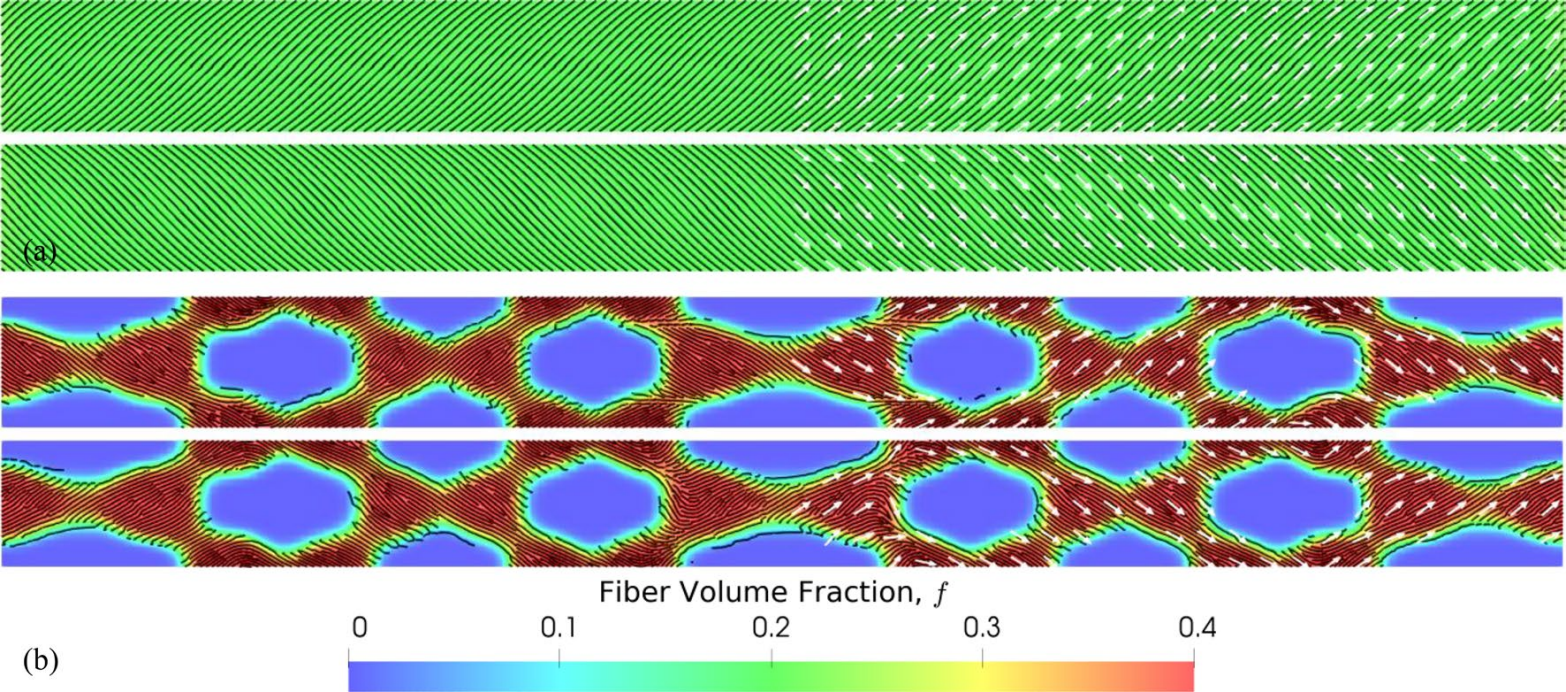
Optimization of a laminated composite cylindrical tube: optimized distribution of fiber volume fractions, $f$ (color) and orientations, $\theta$ (white arrows) for (**a**) continuous $f$ and (**b**) discrete $f$ problem cases. The black curves show the fiber layout obtained from stripe patterns algorithm. In each case, only a quarter of the structure between xz- and yz-planes for both inner (bottom) and outer (top) layers are shown.

Figure 5a,b, respectively, show the optimized designs obtained with continuous and discrete variation of $f$ with the spacing between adjacent fibers set to 1.2 mm at $f=0.4$. With the continuous case, we obtained a result with nearly uniform $f$ distribution and fibers oriented along $\pm$ 45°. The fiber layout results in a spiral arrangement identical to filament wound composites that are known to be optimal. The discrete case resulted in a truss-like embedding of composite material with $f=0.4$ surrounded by the matrix ($f=0$), similar to results obtained with multi-material SIMP-based topology optimization^97^.

### Spherical structure

**Figure 6:**
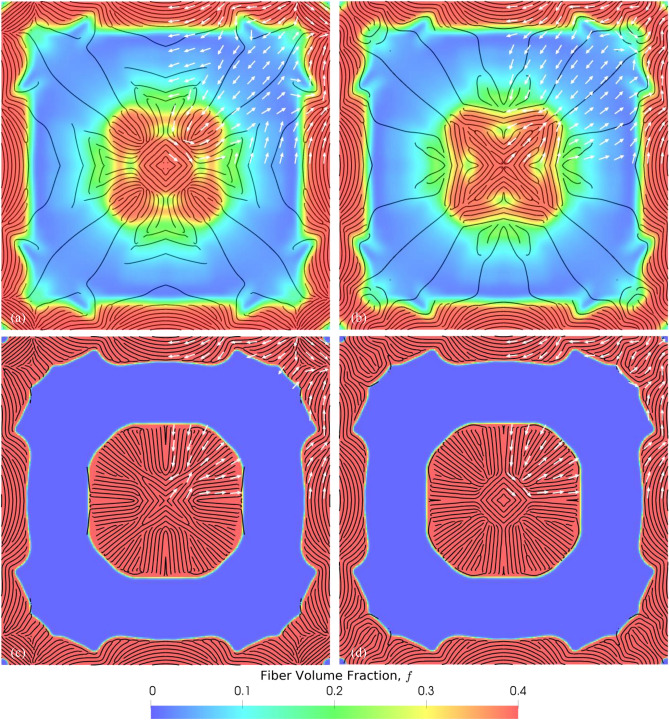
Optimization of a laminated spherical structure: optimized distribution of fiber volume fractions, $f$ (color) and orientations, $\theta$ (white arrows) for (**a**,**b**) continuous $f$ and (**c**,**d**) discrete $f$ problem cases. (**a**,**c**) correspond to the inner layer and (**b**,**d**) to the outer layer of the composite. The black curves show the fiber layout obtained from stripe patterns algorithm.

We optimized a 2-layered composite structure with the geometry of a truncated spherical shell of thickness 1 mm that could find application as a roof in architecture (see Fig. 4c,d). The surface used was obtained by truncating a sphere of radius 2 m (center at origin) with four planes defined by $x=\pm$ 1 m and $y=\pm$ 1 m. The resulting structure projects a square of length 2 m onto the *xy*-plane as shown in Fig. 4c. Each lamina was assumed to be 0.5 mm in thickness and a downwards bending load of magnitude 1 kPa was applied to the center of the structure as shown in Fig. 4c. A length of 0.2 m (on *xy*-plane) of each edge near each corner (see Fig. 4c) was clamped. We took advantage of the fourfold symmetry of the boundary and load conditions and simulated only a quarter of the model. Simultaneously, we imposed on the design fourfold rotational symmetry and reflection symmetry about the diagonal plane. The optimized designs with both continuous and discrete distribution of volume fraction, $f$ are shown in Fig. 6 where the color represents the local $f$ and the fiber orientation at sampled locations is represented by arrows. The fiber layout for the inner and outer layers are also shown in Fig. 6a (c) and 6b (d) respectively for the continuous (discrete) variation case. We set the fiber spacing to 2.54 cm when $f=$ 0.4. Both continuous and discrete cases show similar placement of the fibrous material along the structural boundaries and the central loaded region. In these regions, the bending stress caused by the applied load is maximum. The two fiber rich regions in each case are separated by low stiffness material which in the discrete case is the pure matrix and in the continuous case, low volume fraction composite.

### Hyperbolic paraboloid structure

**Figure 7:**
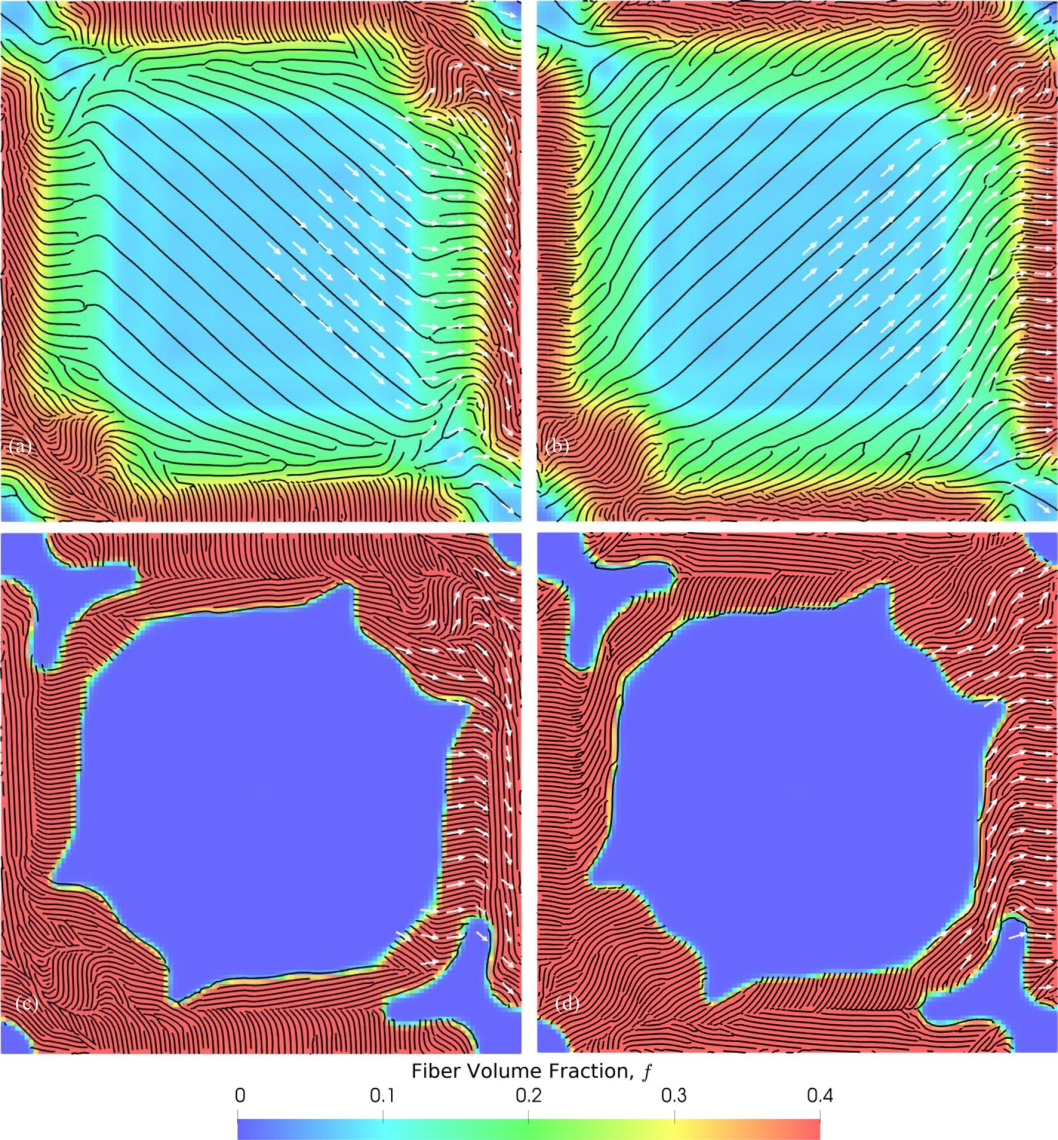
Optimization of a laminated hyperbolic paraboloid structure: optimized distribution of fiber volume fractions, $f$ (color) and orientations, $\theta$ (white arrows) for (**a**,**b**) continuous $f$ and (**c**,**d**) discrete $f$ problem cases. (**a**,**c**) correspond to the inner layer and (**b**,**d**) to the outer layer of the composite. The black curves show the fiber layout obtained from stripe patterns algorithm.

A hyperbolic paraboloid is a non-developable, doubly ruled surface with a negative Gaussian curvature everywhere (see Fig. 4e–g). We optimized a hyperbolic parabolic shaped shell structure, common in architecture, to demonstrate the generality with which our workflow can be applied to any arbitrary surface. The structure was obtained by truncating the surface $xy=$ 2.5 with four planes given by $x=\pm$ 5 m and $y=\pm$ 5 m and imparting it a thickness of 20 mm. The structure was assumed to be clamped at two diagonally opposite corners as shown in Fig. 4e and subjected to a uniform traction of magnitude 100 kPa acting over the entire surface in the direction of *z*-axis. To simplify the optimization problem, we imposed design symmetry about two planes that are perpendicular to the *xy*-plane and oriented along $\pm$ 45° to the *x*-axis. The results of the continuous and discrete cases are shown in Fig. 7. As before, the colors indicate the local $f$ and the arrows indicate the local orientation, $\theta$. The angles are only shown for a quarter of the model and are symmetric about the prescribed symmetry planes. The fiber spacing was set to be 0.1 m at $f=$ 0.4. Just as with the spherical shell case, the continuous (Fig. 7a,b) and discrete (Fig. 7c,d) cases have fiber rich boundary regions. The central region is matrix for the discrete case owing to the restriction of $f$ to either 0 or 0.4, while it is a composite with low $f$ for the continuous case. The fiber orientations in the lower (Fig. 7a,c) and upper (Fig. 7b,d) layers have a difference of about 90° in both cases.

## Conclusions

In this paper, we presented a complete design to manufacture workflow for advanced manufacturing of structures made of architected materials and specialized it for laminated continuous fiber reinforced composites. The design aspect of the workflow enables simultaneous optimization of both the macroscale topology and the spatially varying microstructure, thus blurring the boundary between structure and material. This is one of the first multiscale TO design approaches to specialize in 2.5D structures in the form of laminated variable stiffness continuous FRCs. We connected our design approach to digital voxel-based fabrication through the novel stripe patterns algorithm, thus making this one of the few fully digital design to manufacture workflows in the literature. We demonstrated the unique capability of the stripe patterns algorithm in realizing curved fiber layouts with both uniform as well as non-uniform volume fractions. The stripe patterns algorithm and thus the workflow can be extended to other interesting microstructure topologies (e.g., square and triangular lattices). Our approach can be extended to other advanced manufacturing technologies like AFP (see Section 4, SI), but these will likely require the conceptually straightforward modification of the stripe patterns algorithm to incorporate manufacturing constraints such as a maximum physically obtainable fiber curvature.

We demonstrated capabilities of the workflow that are relevant to fiber composites with various laminated FRC structural geometries, viz., plane, cylinder, sphere, and hyperbolic paraboloid to establish the generality of the approach. We employed voxel-based multimaterial jetting to validate the workflow. We chose the planar structures for this exercise to keep things simple and measured the effective structural stiffness in each case. The experimental measurements reasonably agree with the theoretical predictions with the discrepancies arising from a high density of defects i.e., singularities generated during the material compilation step. We showed that the defect density decreases with increasing scale separation (i.e., ratio of characteristic macro- and microscale lengths). Thus, it can be argued that the defects will be a non-issue for typical carbon fiber reinforced composites where the individual fiber diameters (typically < 10 µm) are an order of magnitude lower than the fiber diameters we used (360 µm). At the same time, we can obtain improved results by implementing the following, individually or in combination: (a) constrain the fiber orientation field, $X$, such that field orthogonal to it, ${X}_{\perp }$, is curl free, i.e., $\nabla \times {X}_{\perp }=0$, (b) tailor the stripe patterns algorithm to minimize singularities, and (c) couple the stripe patterns algorithm with the design automation step.

## Supplementary information

Supplementary Information.
