# Supplementary material for: Optimal design and manufacture of variable stiffness laminated continuous fiber reinforced composites
Source: Sci Rep. 2020 Oct 5;10:16507. doi: 10.1038/s41598-020-73333-4 (PMC7536228; doi:10.1038/s41598-020-73333-4)
Supplement: Supplementary file 1 — Supplementary Information. [file 41598_2020_73333_MOESM1_ESM.pdf]

Optimal Design and Manufacture of Variable Stiffness Laminated Continuous Fiber Reinforced Composites

Narasimha Boddeti[1,2*], Yunlong Tang[1], Kurt Maute[3], David W. Rosen[1,4], Martin L. Dunn[5]

[1]Digital Manufacturing and Design Centre, Singapore University of Technology and Design, Singapore

[2]School of Mechanical and Materials Engineering, Washington State University, Pullman, USA

[3]Ann and H. J. Smead Aerospace Engineering, University of Colorado, Boulder, USA

[4]George W. Woodruff School of Mechanical Engineering, Georgia Institute of Technology, Atlanta, USA

[5]College of Engineering, Design and Computing, University of Colorado, Denver, USA

1. Simulations: Setup, Design Evolution and Convergence History

1.1 Topology Optimization

We used SIMP-based multiscale topology optimization method where the macroscale material layout i.e., topology is encoded in the distribution of a normalized fictitious density, ρ. The microstructural parameters of the fiber, viz., fiber orientation, θ and volume fraction, f, provide the microscale design parameters. Given a design problem with a specified design domain, objective and constraints, our multiscale topology optimization method involves an iterative approach with each iteration containing the following steps after a feasible initial design is chosen: 1) Mori-Tanaka homogenization, 2) forward analysis, 3) sensitivity analysis, 4) optimization, and 5) design update. Forward analysis refers to a finite element (FE) analysis step where the homogenized material stiffness tensor from the homogenization step is used to evaluate the design objective (e.g., stiffness, strength) and constraints (e.g., maximum mass or weight) based on the structural response to the given loading and boundary conditions. Sensitivity analysis calculates, using the discrete adjoint method[1], the sensitivity (i.e., gradient) of the objective and constraints to changes in each optimization variable. The optimization step, then, uses these sensitivities to update the optimization variables, s, which are in turn used to update the design parameters ρ, θ and f. We employed the widely used GCMMA method (Globally Convergent Method of Moving Asymptotes) for this[2].

The approach we used in this paper is identical to the one we used in our previous effort where we focused on laminated short-fiber composite structures and the interested readers can refer to it[3]. The only significant difference is that the fibers are assumed to be continuous i.e., fiber aspect ratio, $\alpha \gg 1$ in the current paper.

1.2 Anisotropic Filtering

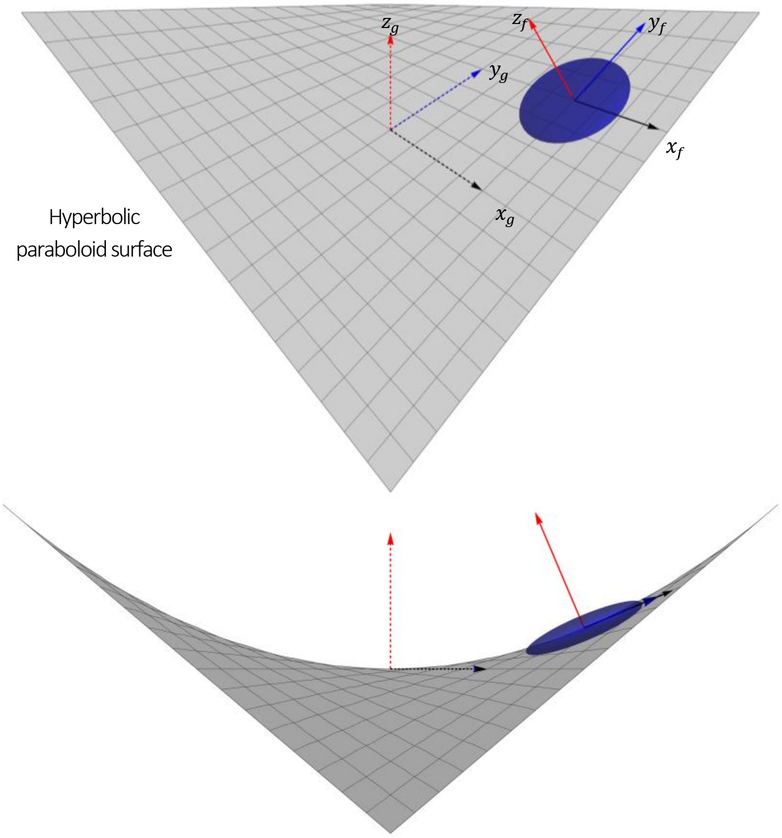

Figure S1 Illustration of anisotropic filtering for the hyperbolic paraboloid shaped laminate. The blue oblate spheroid represents the filtered region. The solid arrows indicate the local coordinate system used to orient the filtered region and calculate the anisotropic filter weights while the dashed arrows indicate the global coordinate system.

Filtering of the optimization variables is known to help regularize the optimization problem[3–5]. The most commonly used filtering approaches include linear[4] and projection filters[5]. Linear filter, in the finite element setting, averages variables defined on FE mesh nodes (optimization variables, s, in our case) within a specified neighborhood, e.g., a sphere or ellipsoid. This helps smooth out undesired large gradients in the design parameters (density, fiber volume fraction and orientation). The projection filter is applied to mitigate the blurring introduced by the linear filter and to obtain a distinct 0-1 design, i.e., no intermediate values making it easier to interpret the resulting design. Projection filter achieves this by mapping the input, typically defined over [0,1], to either 0 or 1 with the help of mathematical functions like smooth-Heaviside step or sigmoid. The linear filter is combined with a projection filter to achieve a minimum length scale when applied to density, ρ or volume fraction, f.[4,5]

In this paper, each design parameter was defined as an elemental property, $\phi = \phi(s_\phi)$ (ϕ could be ρ, θ or f), using a filter, F_ϕ operating on the nodal optimization variables, s_ϕ ($s_\phi \subseteq s$). This filter operator was set to be either a linear filter, $L(s_\phi)$ or a combination of linear and projection filters, $P\left(L(s_\phi)\right)$. Thus:

$$\phi(s_\phi) = \phi_{min} + (\phi_{max} - \phi_{min})F_\phi(s_\phi) \quad \text{(S1)}$$

Here, ϕ_{min} and ϕ_{max} are the bounds set on the design parameters (see Table S1). The linear filter operation is defined as a weighted average of s_ϕ:

$$L(s_\phi, r_s^p, r_s^t) = W s_\phi \quad \text{(S2)}$$

Here, W is a matrix containing weights assigned to neighboring s_ϕ. The parameters r_s^p and r_s^t are the radii of an oblate spheroid (ellipsoid with two of three radii equal) that define the neighborhood over which ϕ is averaged. We set $r_s^p = r_s^t$ for ρ and f, making it a spherical neighborhood, but use different values for θ to decouple ($r_s^p > r_s^t \sim$ layer thickness) the influence of optimization variables across different layers of the laminate. To do this, we transformed the ellipsoidal neighborhood at the point of interest such that the axis with smallest radius, r_s^t gets aligned with the local normal of the surface as shown in Fig. S1. The elements of the matrix W were defined as:

$$\begin{aligned} W_{ij} &= \frac{w_{ij}}{\sum_j w_{ij}} \\ w_{ij} &= max(0, 1 - r_{ij}) \end{aligned} \quad \text{(S3)}$$

Here, w_{ij} are the weights assigned to the pair of ith element and j^{th} node. These depend on a distance measure denoted by r_{ij} and defined as:

$$r_{ij}^2 = \left(\frac{x_i - x_j}{r_s^p}\right)^2 + \left(\frac{y_i - y_j}{r_s^p}\right)^2 + \left(\frac{z_i - z_j}{r_s^t}\right)^2 \quad \text{(S4)}$$

Here $\{x_i, y_i, z_i\}$ are the coordinates of the centroid of element *i* while $\{x_j, y_j, z_j\}$ are the coordinates of node *j*. It is to be noted that the linear filter averages $\theta = -\pi/2$ and $\theta = \pi/2$ to 0 even as $\theta = \pm\pi/2$ result in the same material stiffness tensor.

The projection filter is used to demarcate the different material phases used in the optimization problem. Typically, with isotropic materials, it is applied on linearly filtered ρ. In our case, we applied it on either ρ or f as needed. The projection operator is defined as:

$$P(L) = \frac{tanh(\beta\eta) + tanh(\beta(L - \eta))}{tanh(\beta\eta) + tanh(\beta(1 - \eta))} \quad \text{(S5)}$$

Here, β and η are parameters that control the sharpness and threshold of the filter, respectively. Based on our bounds for s_ϕ, L varies between ϵ and 1, i.e., $0 \le \epsilon \le L \le 1$ where $\epsilon = 0.001$ for ρ and 0 for f. Values

of L below η are mapped to 0 and above to 1. To avoid numerical issues related to the large gradients associated with this filter, we increased β from 0 to 50 and decrease η from 0.5 to 0.1 every 100 iterations in four steps.

1.3 Simulations: Setup

Table S1 Topology optimization problem setup: design domain, constraint, design parameters and filter parameters

Problem	*Dimensions (cm× cm× cm)*	*Constraint*	*Design Parameter Bounds*	*Filter Parameters*	
				Linear Filter Radii (cm)	*Projection Filter Applied?*
Plate with a hole	$8 \times 4 \times 1$	$V_f \leq 0.05 V_\Omega$	$\rho = 1$	Not applicable (n.a.)	
			$0 \leq f \leq 0.1$	$r_s^p = r_s^t = 0.1$	No
			$-\pi/2 \leq \theta \leq \pi/2$		No
Laminated Plate	$10 \times 10 \times 0.77$	$V \leq 0.5 V_\Omega$	$0.001 \leq \rho \leq 1$	$r_s^p = r_s^t = 0.8$	Yes
			$f = 0.1$	n.a.	
			$-\pi/2 \leq \theta \leq \pi/2$	$r_s^p = 0.4$<br>$r_s^t = 0.1$	No
Cylindrical Tube	$50 \times 6 \times 0.2$ (Bounding box)	$V_f \leq 0.05 V_\Omega$	$\rho = 1$ (continuous)<br>$\rho = f/f_{max}$ (discrete)	n.a.	
			$0 \leq f \leq 0.4$	$r_s^p = r_s^t = 2$	No (cont.)<br>Yes (disc.)
			$-\pi/2 \leq \theta \leq \pi/2$	$r_s^p = 1$<br>$r_s^t = 0.25$	No
Spherical Structure	$100 \times 100 \times 58.9$ (Bounding box)	$V_f \leq 0.05 V_\Omega$	$\rho = 1$ (continuous)<br>$\rho = f/f_{max}$ (discrete)	n.a.	
			$0 \leq f \leq 0.4$	$r_s^p = r_s^t = 6.4$	No (cont.)<br>Yes (disc.)
			$-\pi/2 \leq \theta \leq \pi/2$	$r_s^p = 6.4$<br>$r_s^t = 0.05$	No
Hyperbolic Paraboloid Roof	$1000 \times 1000 \times 500$ (Bounding box)	$V_f \leq 0.05 V_\Omega$	$\rho = 1$ (continuous)<br>$\rho = f/f_{max}$ (discrete)	n.a.	
			$0 \leq f \leq 0.4$	$r_s^p = r_s^t = 80$	No (cont.)<br>Yes (disc.)
			$-\pi/2 \leq \theta \leq \pi/2$	$r_s^p = 40$<br>$r_s^t = 0.1$	No

As stated in the main text, the objective in all the topology optimization problems is to maximize stiffness. This was achieved by minimizing the resulting strain energy in the structure due to the applied load and boundary conditions. A constraint on either overall material usage or fibrous material usage was imposed. Tables S1 tabulates the specific constraint used for each problem along with design parameter bounds and anisotropic filter parameters. In Table S2, we list the symmetry conditions imposed on each design parameter.

These symmetry conditions were imposed explicitly for the cylinder and hyperbolic paraboloid problems where a full model was used for FE simulations. For the other problems, we used only a quarter of the design domain for FE simulations while assuming a symmetric structural response. Thus, most of the symmetry conditions were imposed implicitly. The symmetry about the diagonal plane for the plate and spherical shell problems were imposed explicitly. For all the laminated structures considered, we used hexahedral solid shell elements based on Schwarze and Reese's work[6].

Table S2 Topology optimization problem setup: imposed symmetry conditions

Problem	*Design Parameters*	*Symmetry Conditions*
Plate with a hole	ρ	$\rho(x,y)=1$
	f	$f(x,y)=f(-x,y)=f(-x,-y)$
	θ	$\theta(x,y)=-\theta(x,-y)=\pi+\theta(-x,-y)$
Laminated Plate	ρ	$\rho(x,y,z)=\rho(-x,y,z)=\rho(-x,-y,z)=\rho(y,x,z)$
	f	$f(x,y,z)=0.1$
	θ	$\theta(x,y,z)=-\theta(x,-y,z)=\pi+\theta(-x,-y,z)=-\frac{\pi}{2}-\theta(y,x,z)$
Cylindrical Tube	ρ	$\rho(x,y)=1$
	f	$f(x,y,z)=f(-x,y,z)=f(-x,-y,z)=f(y,x,z)=f(x,y,-z)$
	θ	$\theta(x,y,z)=\theta(-x,y,z)=\theta(-x,-y,z)=\theta(y,x,z)=\theta(x,y,-z)$
Spherical Structure	ρ	$\rho(x,y)=1$
	f	$f(x,y,z)=f(-x,y,z)=f(-x,-y,z)=f(y,x,z)$
	θ	$\theta(x,y,z)=-\theta(x,-y,z)=\pi+\theta(-x,-y,z)=-\frac{\pi}{2}-\theta(y,x,z)$
Hyperbolic Paraboloid Roof	ρ	$\rho(x,y)=1$
	f	$f(x,y,z)=f(y,x,z)=f(y,-x,z)$
	θ	$\theta(x,y,z)=\theta(y,x,z)=\theta(y,-x,z)$

1.4 Simulations: Thickness Optimization

The anisotropic filtering scheme enables us to optimize thickness of the laminate along with the microstructure. To demonstrate this, we set up an identical problem to the laminated plate optimization problem (see Fig. 3, main text) with only one difference. We modified the linear filter applied to densities to an anisotropic one with $r_s^p = 0.4$ mm and $r_s^t = 0.1$ mm. The reduced filter radii have the effect of reducing the smoothening effect of the linear filter and enables appearance of thinner features locally, more so in the thickness direction. This manifests as an ability to optimize thickness as can be seen in Fig. S2. It is to be noted that the resulting thickness variation is continuous and hence results in thin sections where fibers cannot be placed with the fabrication technology we used. Hence, the design with isotropic density filtering was preferred in the current study.

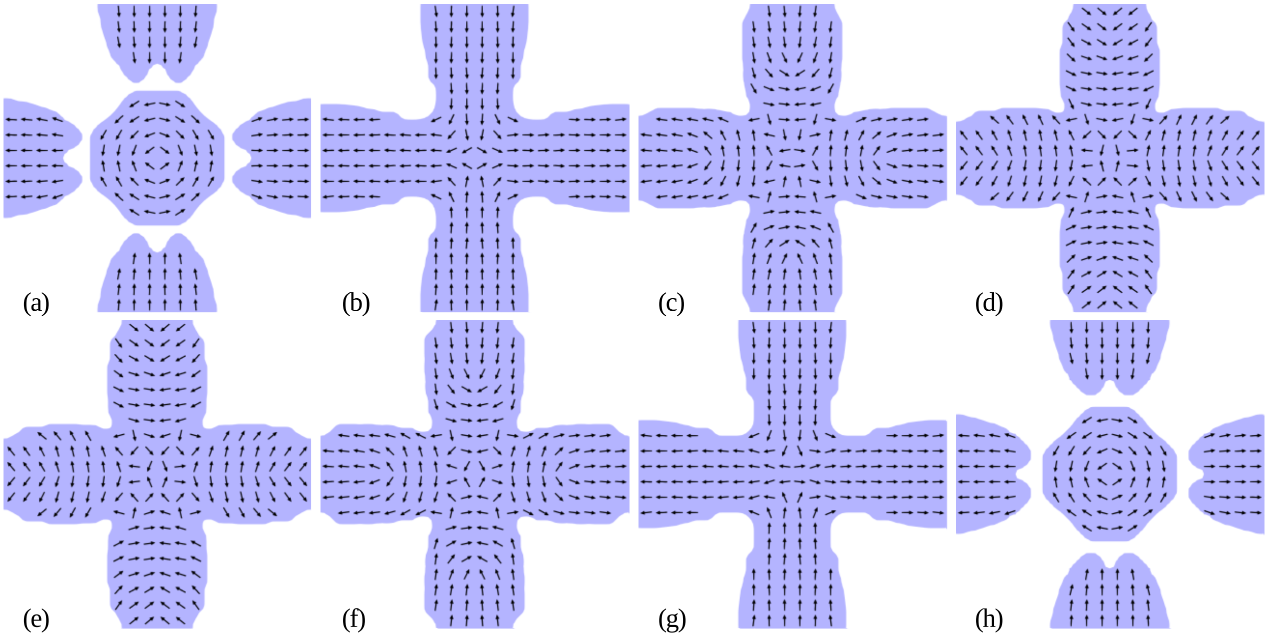

Figure S2 Thickness optimization of a laminated plate with an anisotropic density filter: (a-h) show the macroscale design for layers 1 to 8 respectively. Also seen are the optimized fiber orientations at sampled locations.

1.5 Simulations: Convergence

Figure S3 shows the initial, intermediate and final optimized designs for the design optimization of a composite plate with a hole, while Fig. S5 does the same for the laminated plate. The plots of objective and constraint values for the planar problems are shown in Fig. S4, while Figs. S7-S9 show the same for non-planar problems. For the problems where the projection filter was employed, the effect of the change in the filter parameters, β and η, can be clearly seen as spikes in the constraint graph (or dips in the objective graph) at 100th, 200th, 300th and 400th steps (see plots on the right in Fig. S3 and Figs. S7-S9). In all the optimization problems, we normalized the objective with the strain energy of the initial design and chose an initial design with a uniform f or ρ that satisfied the constraint. The initial fiber orientations were, however, chosen through trial and error to avoid local minima. We considered the optimization problem to have converged when the constraint was satisfied and the change in the objective value between two consecutive design iterations stays below 1e-4 for ten consecutive iterations. In all the cases, we ran the optimization procedure past the point of convergence until the algorithm reached a set maximum number of iterations (400 for problems without projection and 800 with projection). Interactive three-dimensional visualizations of the designs are available on the author's webpage: https://m3lab.github.io/cont_fibers.html.

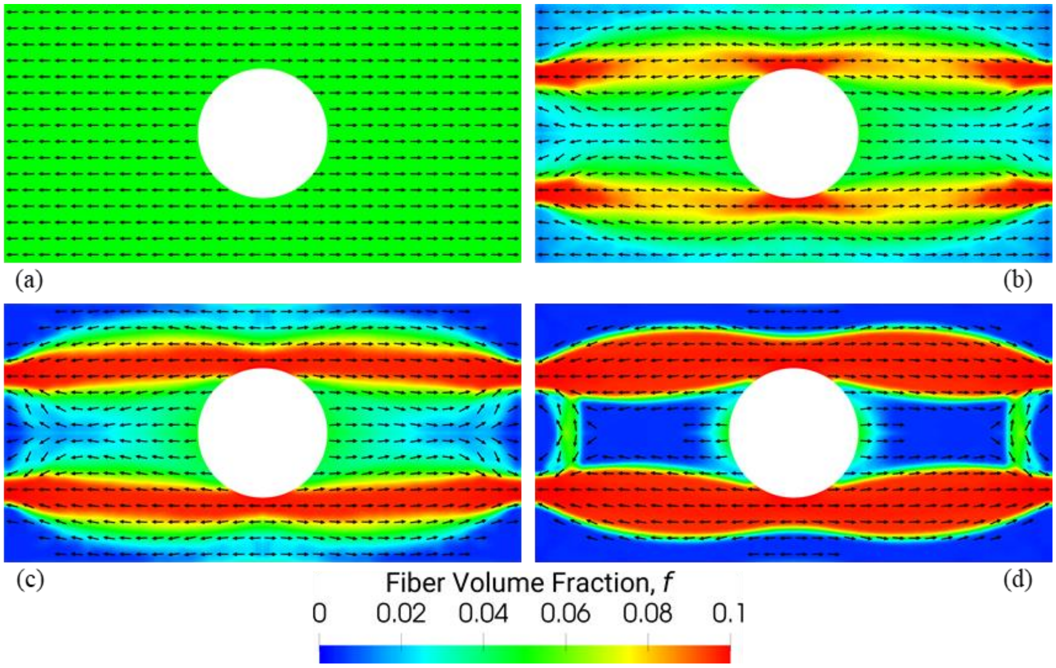

Figure S3 Designs at different stages of the optimization: (a) Initial design (0th iteration), (b) Intermediate design (25th iteration), (c) Intermediate design (50th iteration), and (d) Final design (400th iteration). The colors and arrows indicate the local fiber volume fraction (legend at the bottom) and orienation respectively.

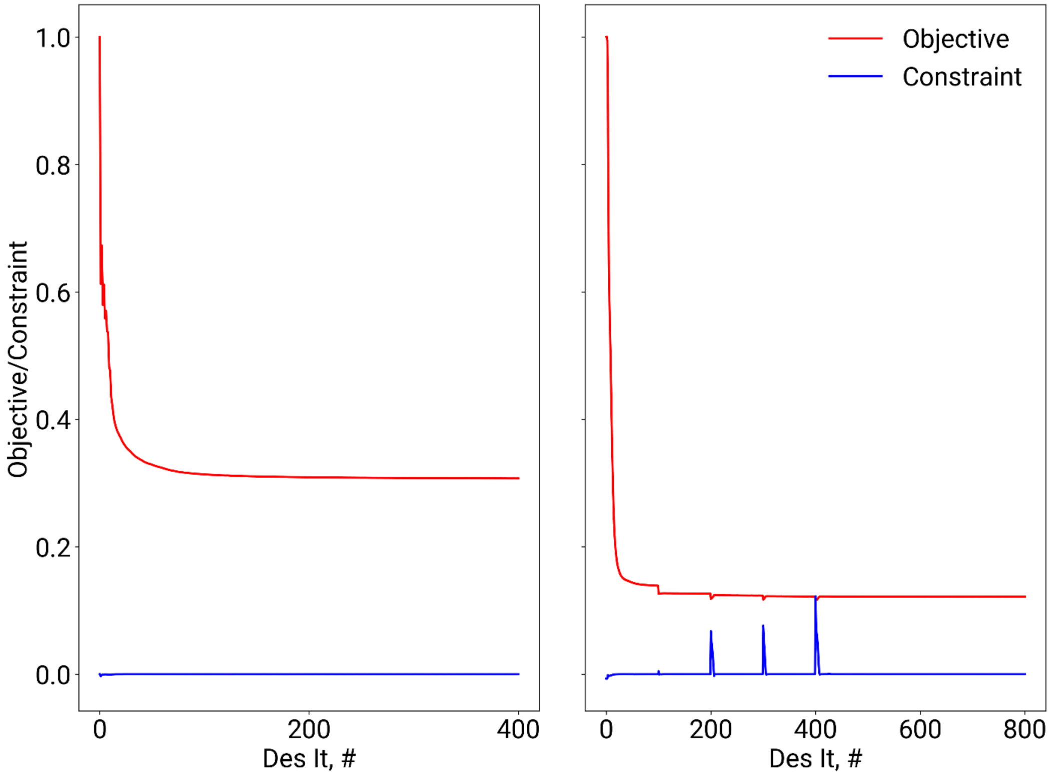

Figure S4 Objective and constraint histories for the planar problems, plate with a hole (left) and laminated plate (right)

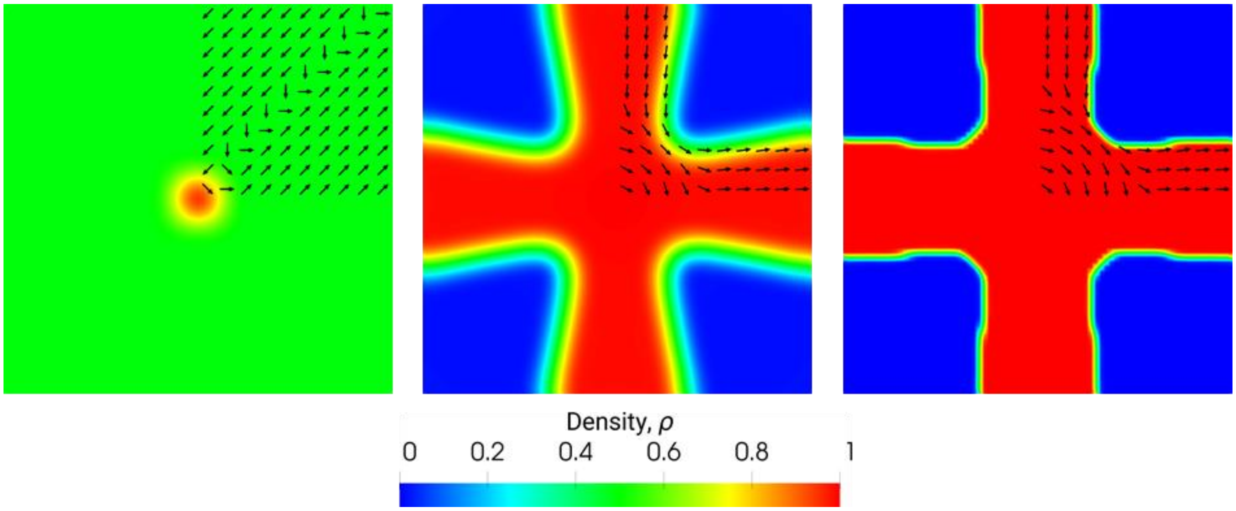

Figure S5 Laminated plate optimization: Initial (0th iteration, left), intermediate (50th iteration, middle) and final designs (800th iteration, right). The color and arrows indicate the local normalized density and orienations for the 1st layer.

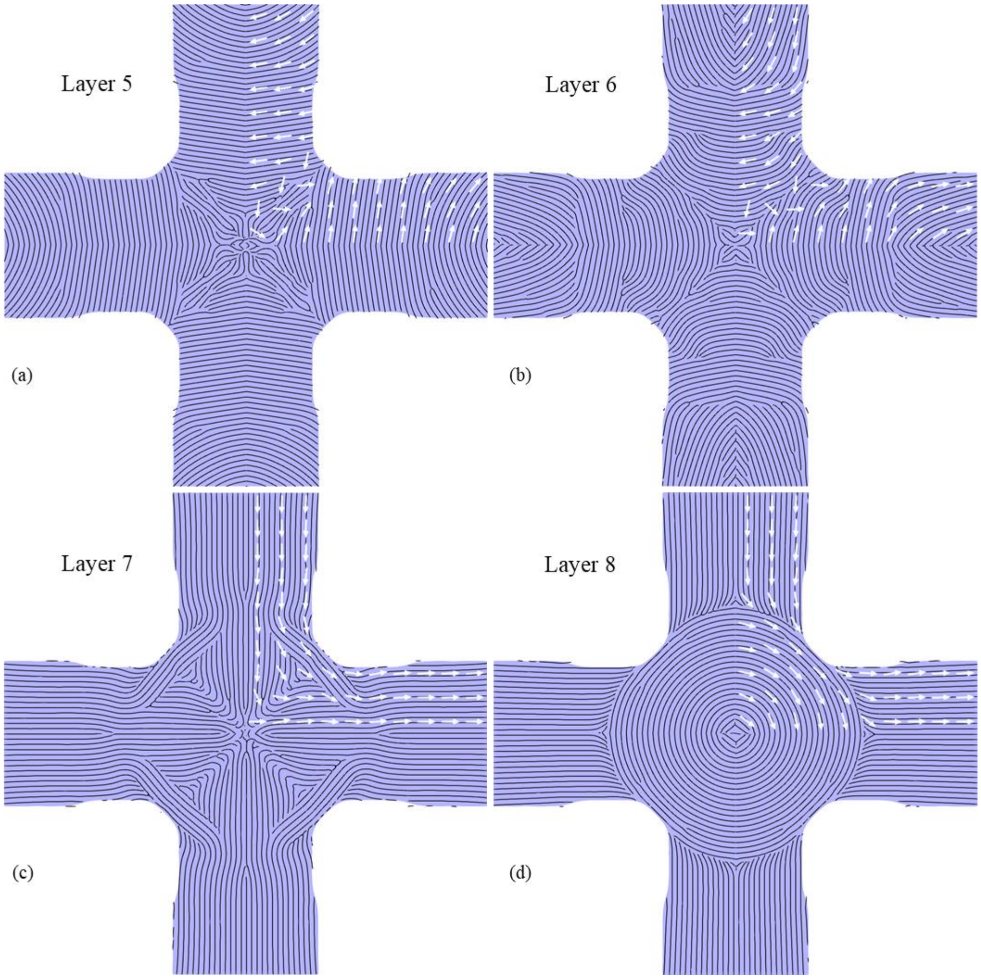

Figure S6 Laminated plate: optimized macroscale design and fiber microstructure for layers 5-8 (layers 1-4 in Fig. 3 of main text)

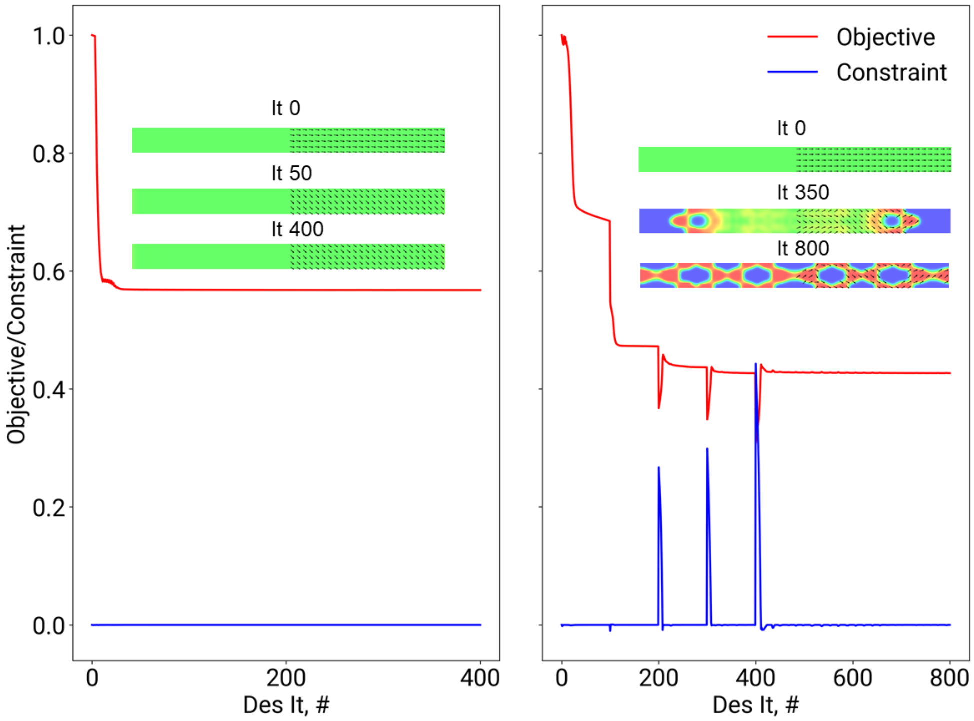

Figure S7 Objective and constraint history for the cylinder problem, with continuous f (left) and discrete f (right). The initial, intermediate and final designs are also shown with the colors and arrows representing the local f and θ of the inner layer.

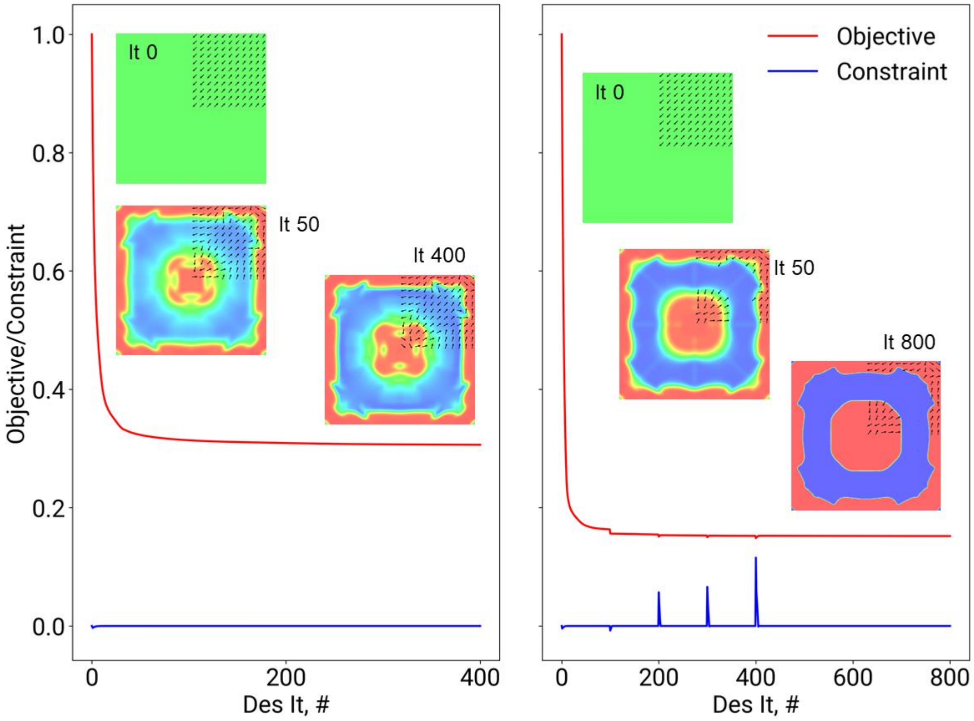

Figure S8 Objective and constraint history for the truncated spherical shell problem, with continuous f (left) and discrete f (right). The initial, intermediate and final designs are also shown with the colors and arrows representing the local f and θ of the inner layer.

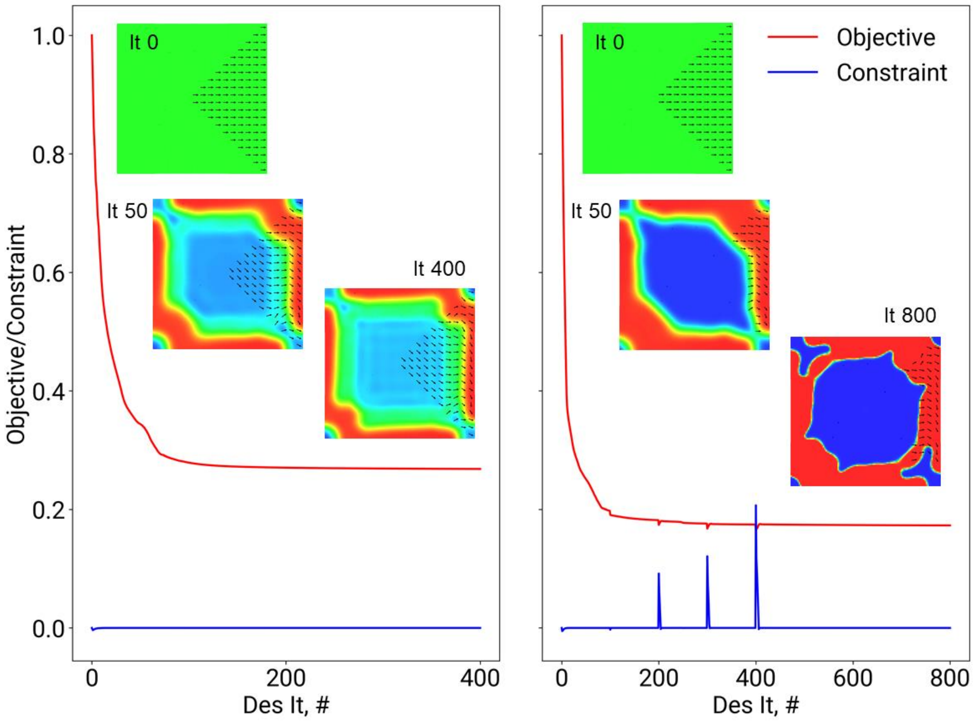

Figure S9 Objective and constraint history for the hyperbolic paraboloid problem, with continuous f (left) and discrete f (right). The initial, intermediate and final designs are also shown with the colors and arrows representing the local f and θ of the inner layer.

2. Material Compilation

We present here the key mathematical details of Knöppel et al.'s[7] stripe patterns algorithm. For a complete and detailed treatment, the readers are referred to Knöppel et al.'s[7] paper. To reiterate from the main text, the goal of the stripe patterns algorithm is to obtain a global, periodic scalar field or *parameterization*, ξ aligned with an input unit vector field X. Mathematically, this is equivalent to finding a scalar field, ξ such that its gradient, $\nabla\xi = \nu X_\perp$, with ν being the prescribed frequency of the stripe periodicity and $X \cdot X_\perp = 0$. Assuming $\xi = \arg(\psi)$ (ψ is a complex valued function with a unit L^2-norm, i.e., $\|\psi\| = 1$), this condition is transformed to:

$$\nabla\psi = i\nu X_\perp \psi \tag{S6}$$

Use of the $\arg$ function ensures the periodicity of ξ. In the discrete setting, Eq. S6 can be easily integrated along an edge of the mesh elements (e_{ij} = edge vector connecting vertices i and j):

$$\int \frac{\nabla\psi}{\psi} \cdot de_{ij} = \int i\nu X_\perp \cdot de_{ij} \tag{S7}$$

The LHS of Eq. S7 can be simplified to $\int \nabla \ln(\psi) \cdot de_{ij} = \ln\left(\frac{\psi_j}{\psi_i}\right)$ by way of the fundamental theorem of calculus for line integrals. Denoting, $Z = \nu X_{\perp}$ and assuming Z varies linearly along the edge simplifies RHS in Eq. S7 to $\frac{1}{2}\left(Z_i \cdot e_{ij} + Z_j \cdot e_{ij}\right) \coloneqq \omega_{ij}$. Thus:

$$\psi_j = e^{i\omega_{ij}}\psi_i \tag{S8}$$

Eq. S8 suggests that the argument of ψ needs to change by ω_{ij} going from vertex i to j for the parametrization to align with the input vector field. Since this condition cannot be satisfied exactly for all the edges, the L^2 error, $E(\psi)$ is minimized. This is defined as:

$$E(\psi) = \sum_{all\ edges} \zeta_{ij}\left|\psi_j - e^{i\omega_{ij}}\psi_i\right|^2 \tag{S9}$$

Here, ζ_{ij} are edge weights that account for the geometry of the mesh elements. For triangular meshes, $\zeta_{ij} = \frac{1}{2}\left(\cot(\alpha_{ij}) + \cot(\beta_{ij})\right)$ with α_{ij} and β_{ij} being the angles opposite to the edge within the two triangles that share the edge. For boundary edges, the unknown angle is set to $\pi/2$. Necessary adjustments are made for edges and triangles with non-orientable and locally orientable Z (see Section 3.3 of Knöppel et al.[7]).

The energy $E(\psi)$ can be represented in matrix form as, $E(\psi) = \mathrm{x}^T A\mathrm{x}$ where x is a column matrix populated with the real and complex parts of ψ. Likewise, the unit norm condition $||\psi|| = 1$ can be discretized as $\mathrm{x}^T B\mathrm{x}$. Matrix A is symmetric positive semi-definite and has structure similar to discrete mesh Laplacian matrix, while B is a diagonal matrix whose diagonal elements depend on the areas of the triangle (see Section 4.1 of Knöppel et al.[7]). Thus, in the discrete setting, the problem distills to:

$$\min_x x^T A x \quad s.t. \quad x^T B x = 1 \tag{S10}$$

This is equivalent to solving for the smallest eigenvalue, λ and the associated eigenvector of the generalized eigensystem, $A\mathrm{x} = \lambda \mathrm{Bx}$.

The parametrization, ξ is obtained from the solution of Eq. S10 by first extracting ψ. Using $\arg\psi$ directly to obtain ξ at each vertex will lead to severe aliasing on coarse meshes. Thus for each triangle ijk, ξ is calculated at one arbitrarily chosen corner as $\xi_i = \arg\psi_i$ and values at the other two corners are locally adjusted using ω_{ij} and optimal ψ (see Section 4.3 of Knoppel et al). Linear interpolation of ξ over each triangle and use of a periodic color function i.e., ascribing the same color to values separated by $2n\pi$ (n is an integer), then results in density plots as shown in Fig. S10b, S10d and S11b. Iso-$2n\pi$ contours then give the desired fiber layout as shown in Fig. S11c.

Figure S10 demonstrates the stripe patterns algorithm with two simple examples. In the first case, the input unit vector field is $X^1 = \left(\frac{y}{r}, -\frac{x}{r}\right)$ where $r^2 = (x^2 + y^2)$ with x and y being the x- and y-coordinates

respectively. The perpendicular vector field, $X_{\perp}^{1} = \left(\frac{x}{r}, \frac{y}{r}\right)$ has zero curl and thus results in stripe patterns with no singularities except near the origin where the input vector is not well defined (see Fig. S10b). In the second case, the input vector field is $X^{2} = X_{\perp}^{1}$. The curl of the perpendicular vector field is non-zero everywhere which leads to several singularities as can be seen in Fig. S10d.

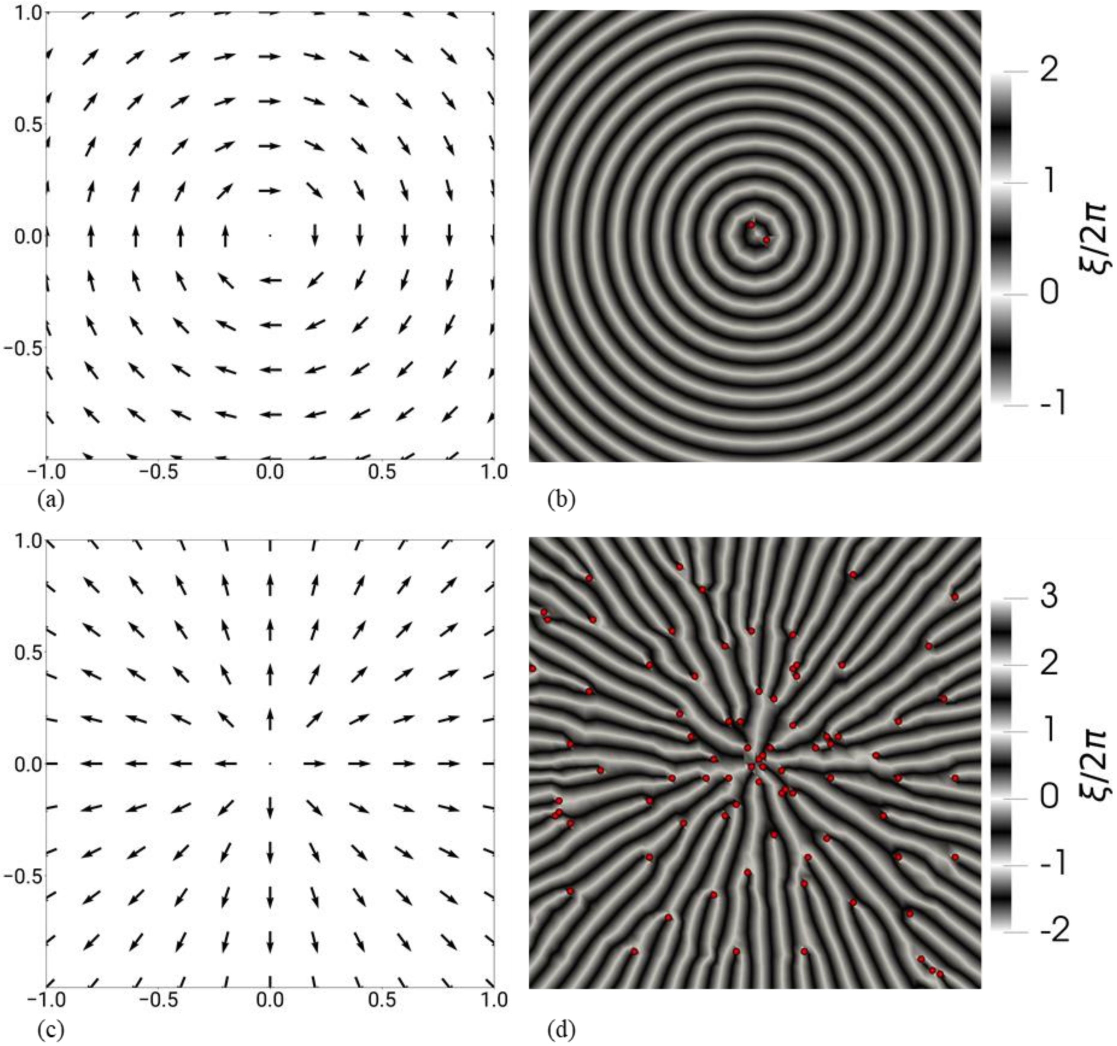

Figure S10 Demonstration of the stripe patterns algorithm: (a, c) Input vector field X and (b, d) ξ from the stripe patterns algorithm. The red points in (b) and (d) indicate singularities.

Figure S11 illustrates the material compilation workflow we adapted in this paper. The workflow begins with the *stripe patterns* algorithm which takes as input the optimized design obtained from the design automation step as an input. To be more specific, the input is a surface mesh with spatially varying stripe frequency, ν and input vector field, X defined at each nodal point. The surface mesh is the mid-plane of the shell elements of each layer used in the FE model (e.g., 8 layers for the laminated plate example, 2 layers for the cylindrical shell). The optimized fiber orientations, θ, on the laminate surface were used to specify the vector field X while stripe frequency ν is defined in terms of the optimized fiber volume fractions, f. To establish the relationship between ν and f, we assumed that the fibers fabricated via material jetting are cylindrical with

radius r_f and are enclosed in imaginary concentric cylindrical tubes that are hexagonally close packed when viewed along the cross-section perpendicular to the local fiber direction. Thus, the diameter of these enclosing cylinders, d_f, gives us the spacing between each fiber and its six equidistant neighbors (assuming uniform packing). The ratio of the cross-sectional areas of the enclosing cylinders and the fiber should then equal their packing densities. The packing density, f_c, for the enclosing cylinders is simply given by $f_c = \pi/\sqrt{12}$ and the packing density for the fiber is f. This then gives us a relation between the fiber spacing, d_f and volume fraction: $d_f = 2r_f \left(\frac{f_c}{f}\right)^{1/2}$. Stripe frequency is then simply $\nu = 2\pi/d_f$. These relationships were found to extend well to non-uniform f.

It is to be noted that the fiber orientations can be flipped by 180° on the laminate surface without affecting the FE simulations. We essentially chose the direction of the vector field, X, to be arbitrary and found it to not affect the stripe patterns and thus the fiber layout significantly. The output of the stripe patterns algorithm is shown in Fig. S11b, where the colors represent the scalar field, ξ. It can be clearly seen that the stripe frequency correlates well with the input volume fractions. In Fig. S11b, we also illustrate the ability to use dissimilar meshes for FE simulations and material compilation. This lets us use fine and coarse meshes for FE and material compilation respectively, thus making the material compilation step fast and computationally efficient. We used extrapolation functions to define X and ν in the non-design domain regions and trimmed them away after the fiber layout was generated as can be seen in Fig. S11c.

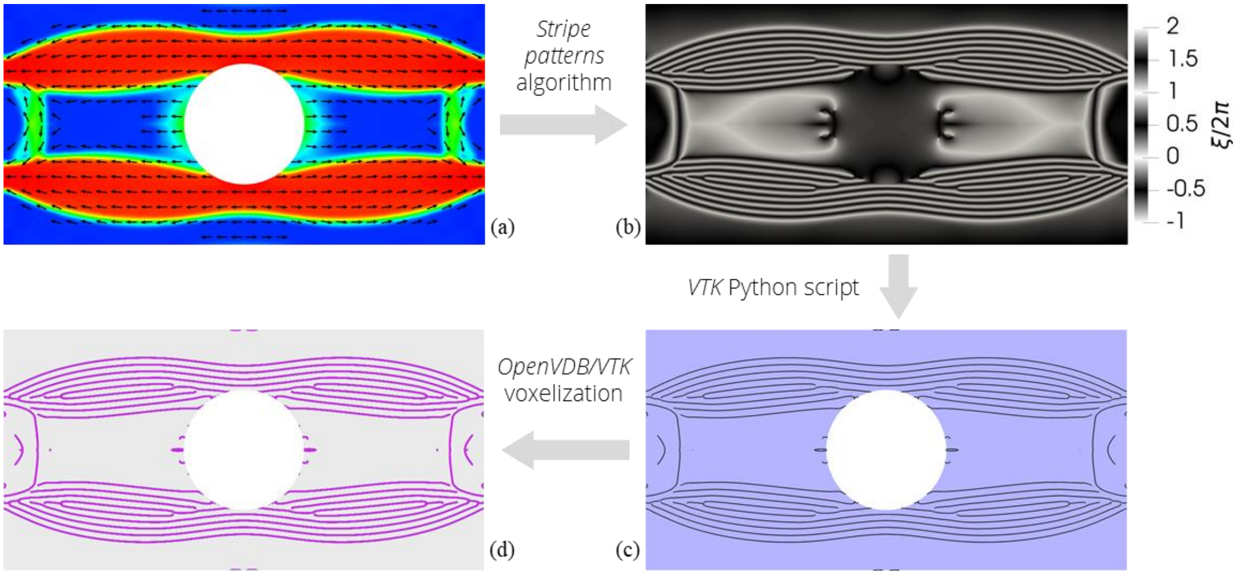

Figure S11 Material compilation workflow: (a) optimized design from the design automatin step with f and θ information forms the input to the stripe patterns algorithm, (b) result of the stripe patterns algorithm with the colors representing the calculated periodic scalar function, α which is stored as a texture coordinate, (c) the fiber layout (black curves) obtained from the texture coordinates with the help of a VTK python script, (d) the 3D voxel model containing the voxelized fibers (magenta) and matrix (light gray) to be used for voxel-based 3D printing

The fiber layout, as shown in Fig. S11c with a single layer of fibers, was obtained with the help of VTK Python wrappings. VTK (visualization toolkit) is a scientific visualization software with several algorithms implemented as filters that act on input datasets to output a modified dataset. These datasets could include a set of points, lines, polygons and polyhedra. In our case, the input dataset is a surface mesh with the calculated

scalar field from the stripe patterns algorithm stored as a texture coordinate. The fiber layout was extracted as iso-contours of the texture coordinate field with the help of *vtkContourFilter*. There is no unique fiber layout, we just choose one among several different sets of contours with the same f and θ distributions that could be obtained from the texture coordinates. For example, the fiber layout in Fig. S11c and Fig. 2d of the main text have the same fiber orientation and volume fraction distribution. The layout in Fig. S11c was obtained by simply offsetting the iso-contour values by half of the fiber spacing value. Other VTK filters were employed to stack the layers of fibers, clean up and save the resulting fiber layout for voxelization. To enable manufacturing with voxel-based 3D printing techniques, a voxel model of the structure to be printed is created with a voxelization routine. Figure S11d shows the top view of the voxel model for a single layer of the composite structure.

A summary of the key steps involved in our material compilation algorithm follows. For each layer of the optimized laminated structure:

1. Prepare a triangulated surface mesh based on the optimized design.
2. Store at each vertex a vector, $Z = \nu/\nu_{max}X$ (X is a unit vector along the optimized fiber orientation). Extrapolate values where needed.
3. Run *stripe patterns* algorithm with the prepared mesh and desired fiber spacing as inputs
    a. Extract edge data: ω_{ij}
    b. Construct energy and mass matrices: A and B
    c. Solve for the smallest eigenvalue of the eigensystem: $A\mathrm{x} = \lambda B\mathrm{x}$
    d. Extract the parametrization, ξ from x
4. Extract iso-$2n\pi$ (or iso-$2n\pi + \delta$, δ is any arbitrary angle) contours

2.1 Algorithm for Connected Fiber Layout

We enumerate here the steps involved in the algorithm for obtaining a connected fiber layout. The input to this algorithm is the disconnected fiber layout comprised of polylines as obtained from the material compilation step.

1. Identify boundary vertices of the polylines, i.e., vertices at the ends that are not shared by two edges of a polyline (vertices on the boundary of the structure are ignored).
2. Identify the closest point, p_n from among the neighboring polylines for each isolated point, p_i and record the distance, d to this point.
3. Identify isolated points that are within a specified threshold distance of a neighboring polyline, i.e., points with $d \leq d_{thr}$ (d_{thr} was set to 1.5 mm, a value of the same order as fiber spacing, d_f).
4. Extend the polyline by connecting p_i and p_n if $d \leq d_{thr}$.

Figure S12a and S12b respectively show the input disconnected fiber layout and the output connected fiber layout generated by this algorithm for the plate with hole structure.

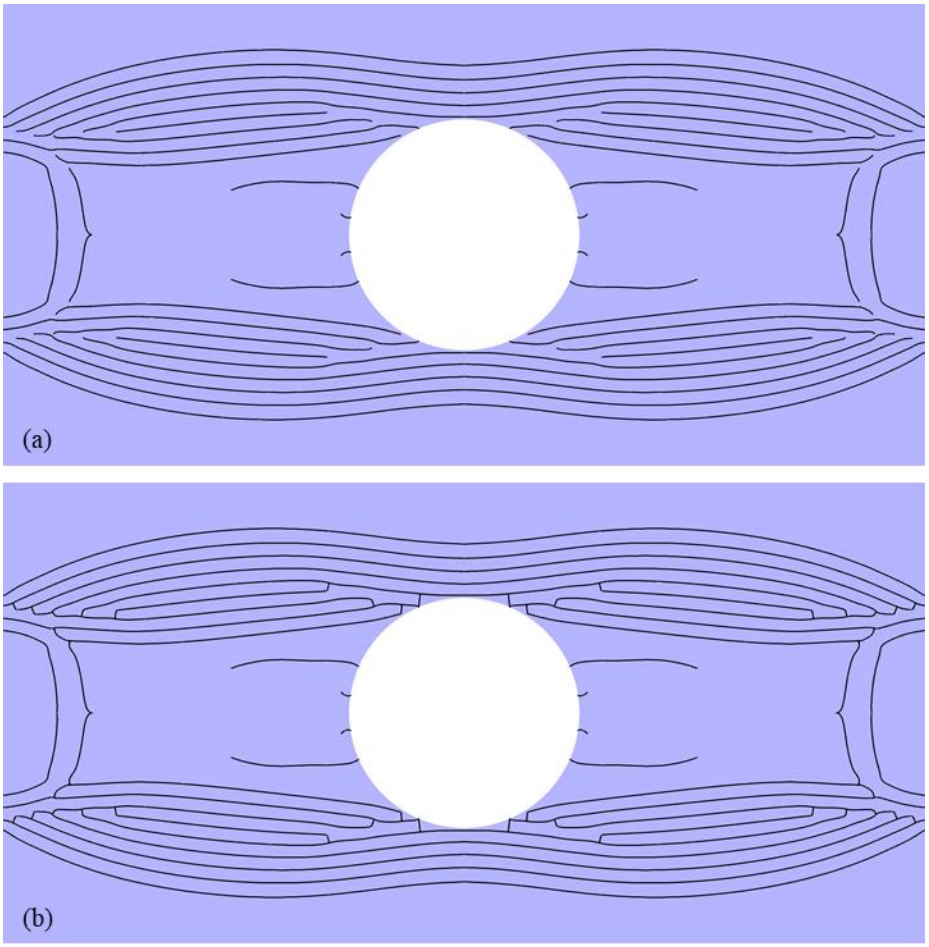

Figure S12 Optimized fiber layout for plate with a hole structure with (a) disconnected and (b) connected fibers.

2.2 Voxelization Procedure

To build voxel models of the fibrous composites, we developed a specialized voxelization routine in C++ employing OpenVDB along with VTK to support file I/O operations. We chose OpenVDB for its highly efficient sparse dynamic data structure and the associated tools for manipulating these data structures which are designed to efficiently store and work with sparse voxel grids. Voxel grids are essentially 3D images with 3D voxels replacing the 2D pixels. Just as in 2D images, voxels store a value which in OpenVDB's case is determined by the voxel location with respect to the geometry being represented by the voxel grid. The voxels within the volume enclosed by the geometry have negative values and the voxels outside positive.

Our routine takes in as input the geometries of the matrix and any supporting structure in the *stl* (stereolithography) format and the fiber layout in VTK's *vtk* or *vtp* format where the fibers are stored as a collection of polylines. Each of the *stl* inputs are then directly converted to OpenVDB voxel grids using OpenVDB's in-built function. An empty voxel grid with a background positive value is initially created for the fibers and the voxels are populated with appropriate values iteratively going through each line segment that make up the fibers. The voxels within a distance of r_f (fiber radius) from each line segment are initialized with a negative value, thus creating the voxelized fiber geometry. We perform CSG (Constructive Solid Geometry) Boolean operations as necessary on the voxel grids such that the voxelized matrix, fiber and

support geometries have no overlaps. The resulting voxel grids can then be used to directly write slices in *bmp* format as required by the 3D printer we used. Alternatively, the voxelized model can be written to a file in VTK's meta image format, *mhd*, for visualization as shown in Fig. S13. In the meta image format, we assign each voxel a unique value to represent the material that occupies the individual voxel. Figure S13a shows the whole 3D voxelized model of a single layer of fibers (magenta) embedded within matrix (light gray), while Fig. S13b shows a slice through the middle of the voxel model.

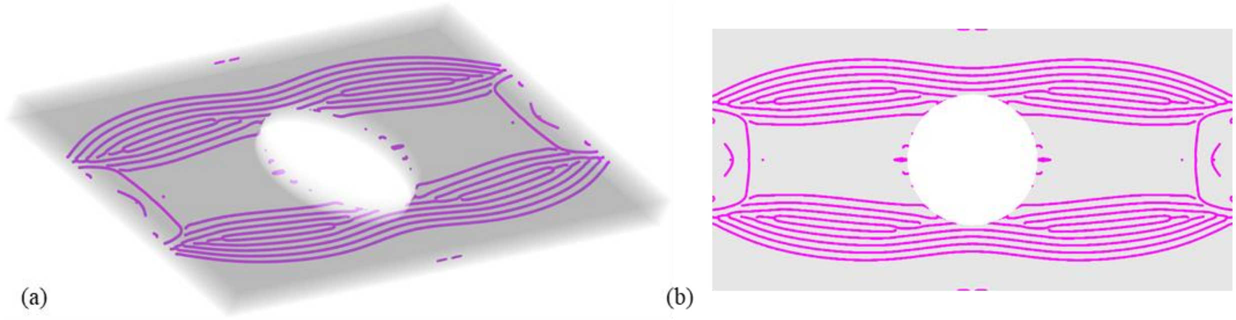

Figure S13 (a) 3D voxel model generated with the OpenVDB/VTK based voxelization procedure, (b) a slice of the voxel model. Magenta and gray represent voxels with fibrous and matrix materials resepectively.

2.3 Material Compiler Validation

To validate the material compilation step, we verified the volume fractions and fiber orientations in the generated fiber layouts for the planar problems. This was achieved with VTK python scripts and the results are shown in Figs. S14-S17. To verify the volume fractions, we randomly sampled 1000 points within the bounding box region of the input geometry. For each point, we calculated the local volume fraction within a $10\times10\times h$ mm cuboidal region ($h=$ laminate thickness). The local volume fraction is given by the ratio of volume of the fibers within the cuboidal region to the volume of the cuboid with consideration for points that lie near the boundaries. The calculated volume fractions are shown in Fig. S14a for the plate with a hole problem and Fig. S16a for the laminated plate structure. Figures S14b and S16b show the deviation of the volume fractions in the generated fiber layout from the optimized values in the form of histograms. It can be clearly seen that we get good agreement in both cases. The mean and standard deviation of the error for the laminated problem are 0.07 and 0.76 while they are -0.38 and 1.5 for the plate with a hole structure. The higher deviation for the plate with a hole structure could be ascribed to spatially varying volume fractions as opposed to uniform volume fractions for the laminated plate.

The geometric representation of the generated fiber layout consists of connected line segments, i.e., collection of polylines. The orientation of the line segments thus gives us the local fiber orientation. By calculating the optimal fiber orientation at the segment's mid-point via interpolation, the accuracy of the fiber orientations in the generated fiber layout can be calculated. We defined a quantity, $\Delta_i = 1-|\mathrm{n_c}\cdot\mathrm{n_o}|$ where $\mathrm{n_c}$ and $\mathrm{n_o}$ are unit vectors along the line segment i and interpolated optimal fiber orientation respectively. This quantity varies between 0 and 1 with zero signaling no error and 1 implies maximum error (i.e., 90° difference). We calculated Δ_i for each segment and found that the *compiled* fiber orientations do not deviate by more than 4° except near defects or singularities as shown in Figs. S15 and S17. It is to be noted that the local material stiffness along the desired fiber direction reduces by 50% when the discrepancy is 4° or more.

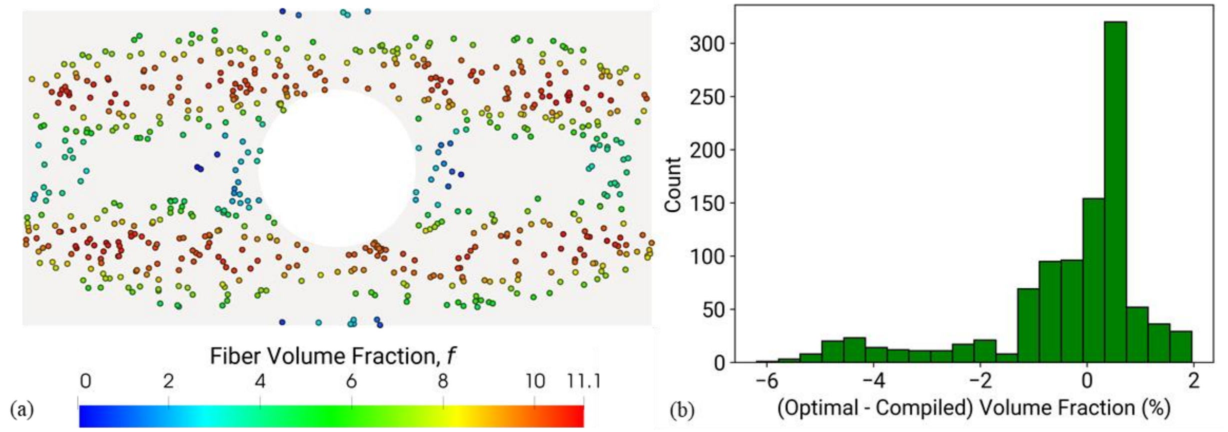

Figure S14 Verification of the volume fractions – plate with a hole (case B). (a) Randomly generated points colored by the calculated fiber volume fractions (points with low f are not shown for visual clarity), (b) histogram showing the deviation of the "compiled" volume fractions from the optimal values.

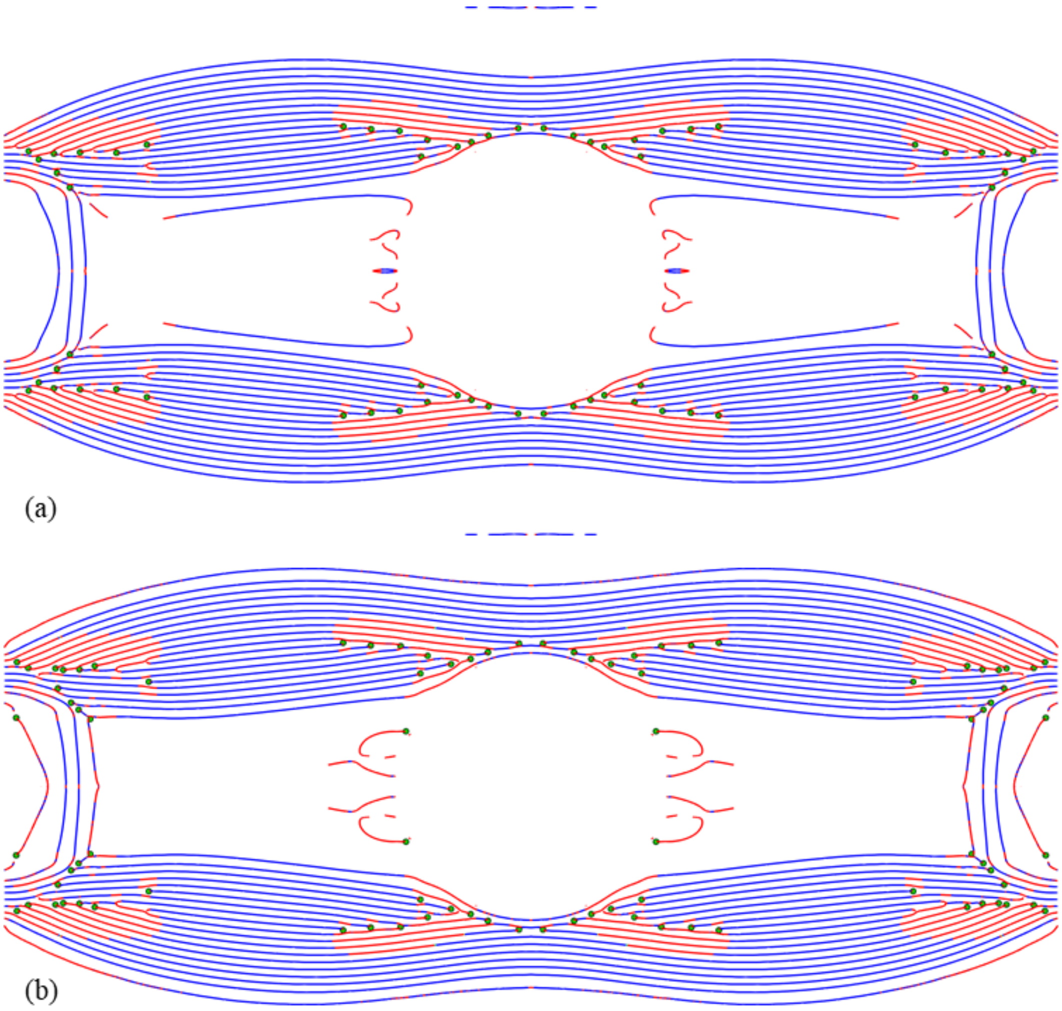

Figure S15 Deviation of the fiber orientations from the desired orientation for the plate with a hole problem (case B). The points are the identified defects while blue and red colors represent segments of the fiber with error in the fiber orientations < 4° and > 4° respectively.

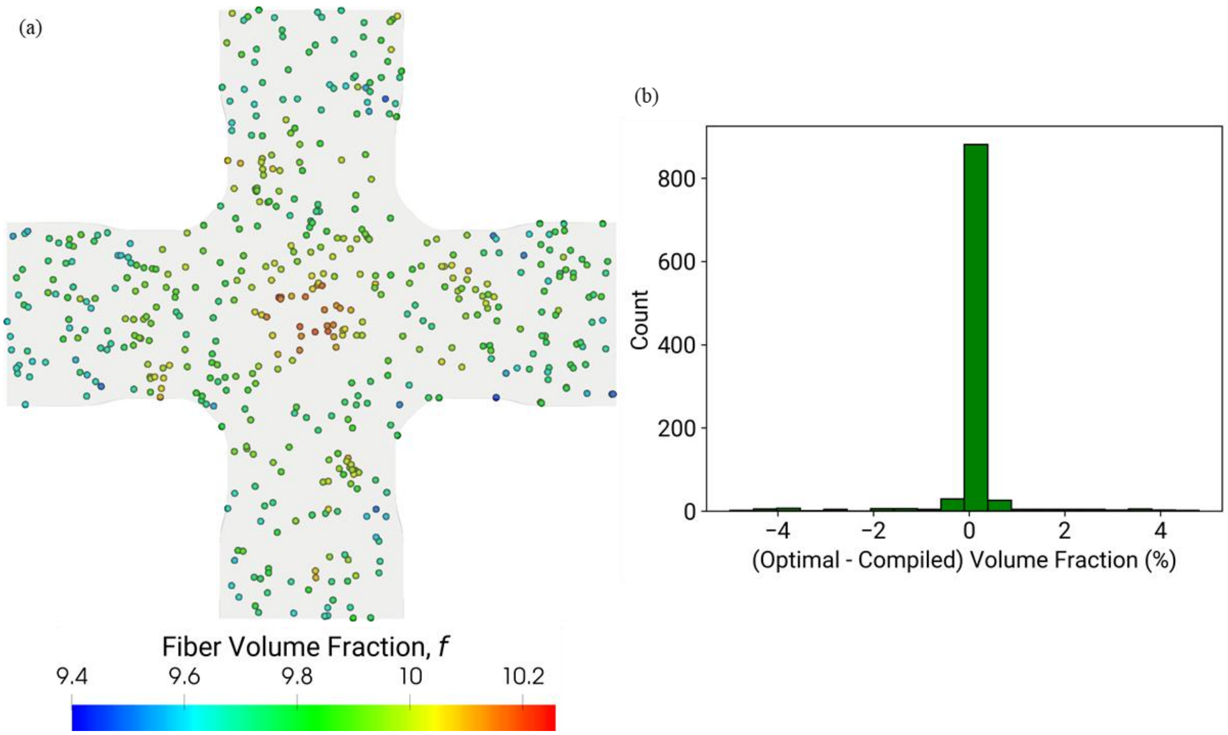

Figure S16 Verification of the volume fractions for laminated plate structure, (a) randomly generated points colored by the calculated fiber volume fractions (points that lie outside the optimized geometry are not shown for visual clarity), (b) histogram showing the deviation of the "compiled" volume fractions from the optimal values.

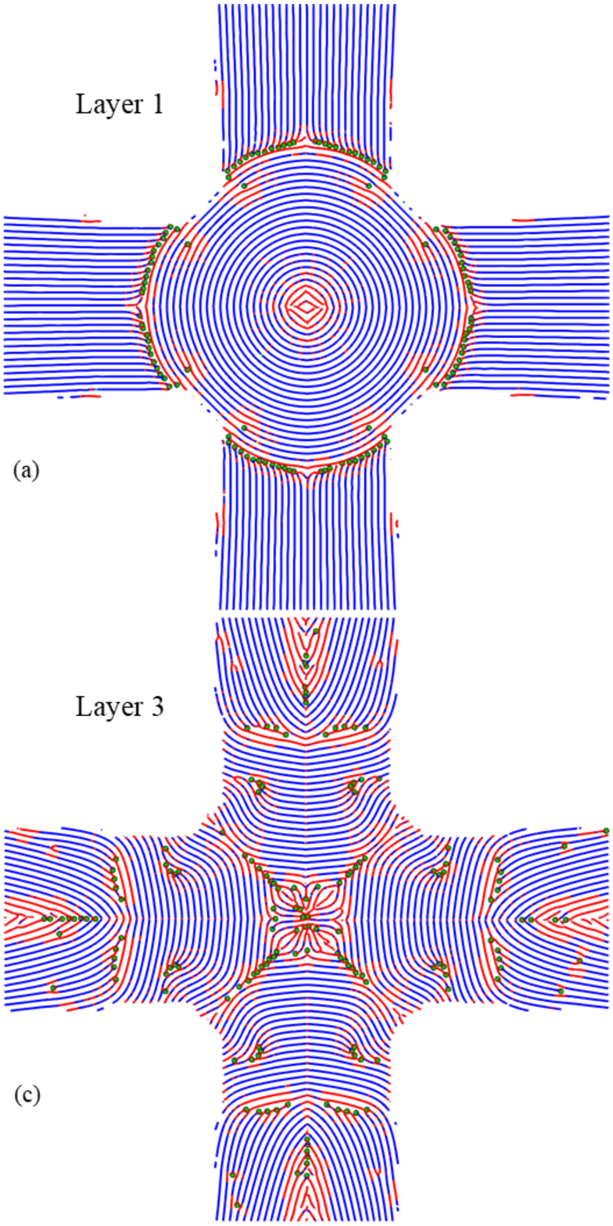

Figure S17 Deviation of the fiber orientations from the desired orientations for the laminated plate structure. The first four layers of the structure are shown with the points identifing defects. The blue and red colors represent segments of the fiber with error in the fiber orientations $< 4^o$ and $> 4^o$ respectively.

3. Experiments

3.1 Material Characterization

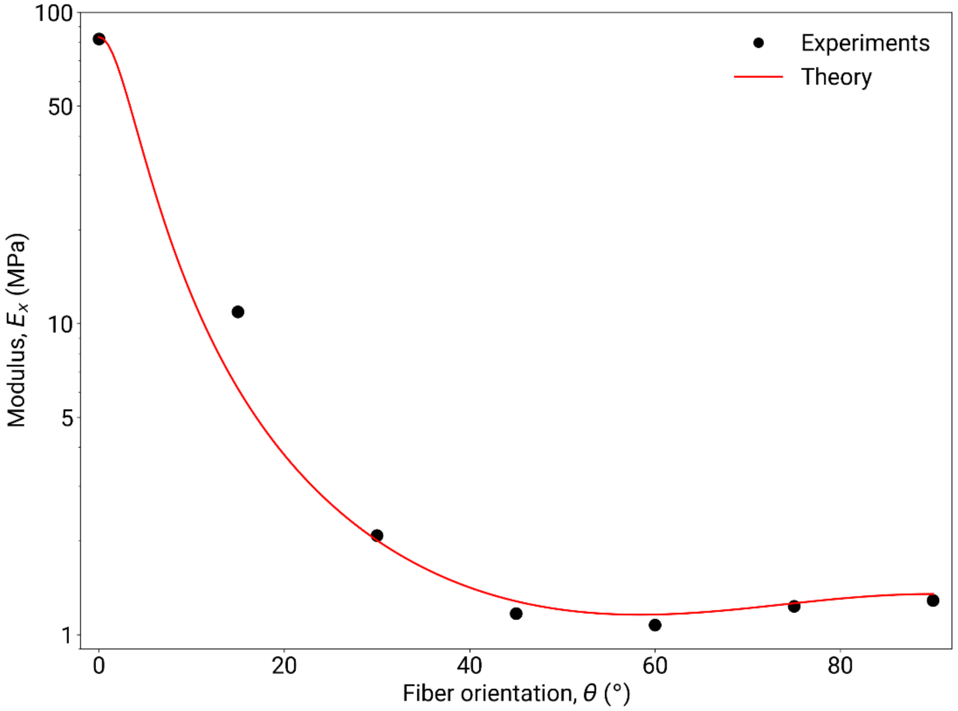

Figure S18 Variation of effective modulus, E_x with fiber orientation, θ of uniaxial tensile testing specimen with the theoretical results obtained from Mori-Tanaka method

To characterize the composite made of the stiff Vero and soft TangoPlus materials, we 3D printed tensile testing samples of length 80 mm and width 20.2 mm on a Stratasys Connex Objet500 multimaterial jetting 3D printer. The samples were 7.7 mm thick such that the volume fraction of continuous Vero fibers embedded in Tango was 10%. We printed seven different samples, each with fibers oriented along a fixed angle with respect to the loading direction which is along the length of the samples. We used the MTS Criterion model 43 universal testing machine with a 1 kN load cell for these tensile tests. The strain applied during the uniaxial tension test was limited to 1% and the strain rate was 1%/min. The effective stiffness modulus along the loading direction for uniaxial tension tests is given by: $E_x(\theta) = \left(\boldsymbol{C}^{\boldsymbol{H}^{-1}}\right)_{11}^{-1}$, where $\boldsymbol{C}^{\boldsymbol{H}^{-1}}$ is the inverse of the homogenized stiffness matrix obtained from the Mori-Tanaka homogenization method. These values for each sample were obtained through a linear fit of the measured stress-strain curves. We used these values to obtain the effective matrix and fiber moduli, E_m and E_f by fitting the experimental data to the Mori-Tanaka method where $\boldsymbol{C}^{\boldsymbol{H}}$ is a function of E_m and E_f. This was done because we found in our previous studies [3,8] that the directly measured matrix and fiber moduli do not predict the composite properties well, possibly a result of *smearing* of the matrix-fiber interface during the printing process. Figure S18 shows the experimentally measured values and the best fit $E_x(\theta)$ according to Mori-Tanaka method. The best fit obtained by minimizing the difference between experimental values and theoretically predicted values with varying E_m and E_f gave us values of $E_m = 0.97$ MPa and $E_f = 0.85$ GPa. These effective moduli were then

used in the design automation step of the workflow for the problems where TangoPlus and Vero were considered.

3.2 Digital Manufacturing and Experimental Validation

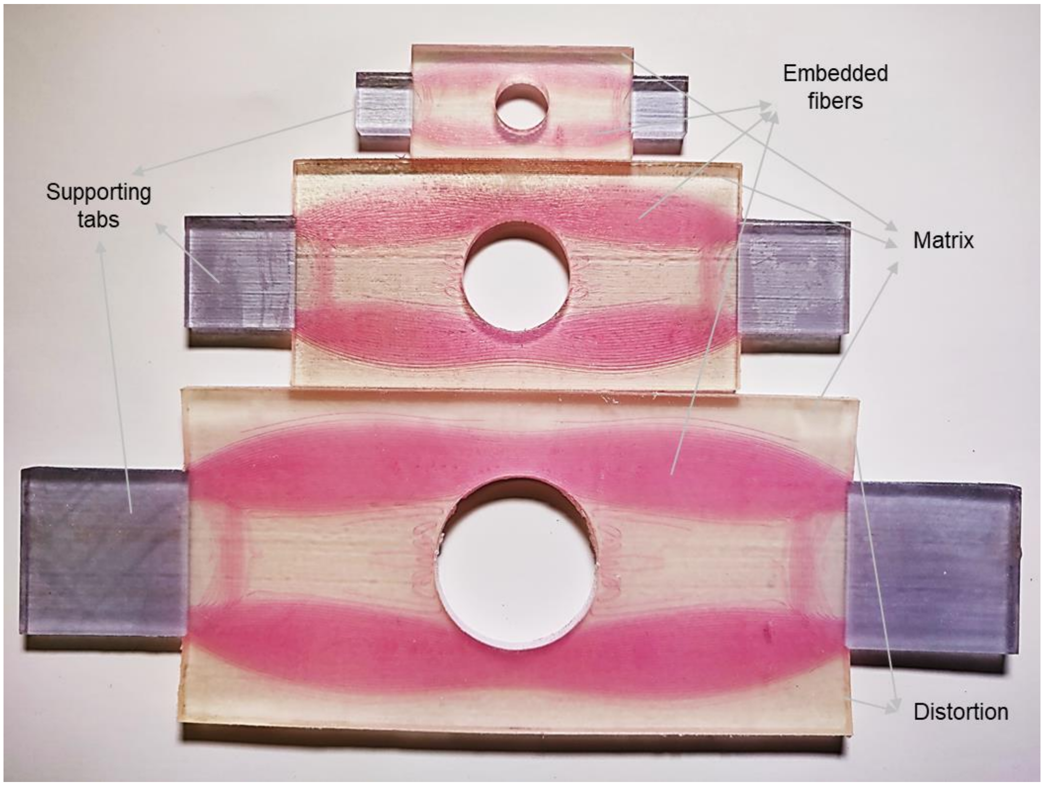

Figure S19 Plate with a hole – 3D printed optimized designs of different sizes (cases A, B and C from top to bottom) with support tabs to facilitate loading

Figures S19 and S20 show the 3D printed planar structures used in our experiments. TangoPlus is known to expand after printing due to strain accumulated during the fabrication process[9,10]. This causes undesired distortion of the printed structure which is visibly apparent in case C given the same thickness but larger size (see Fig. S19).

The optimized structures were printed with relatively rigid tabs (E ~ 1 GPa) to effectively clamp and load the structures. We used the same MTS universal tensile testing machine and 1 kN load cell that we used for material characterization to perform test the printed structures.

For the plate with a hole structure, the tabs were used to clamp in mechanical grips with one of the grips attached to the load cell and the crosshead. The rigid tab also enabled us to impose a uniform displacement load. We specified the rate at which the load was imposed (i.e., crosshead displacement velocity) to be 0.23 mm/min for case A, 0.47 mm/min for case B and 0.7 mm/min for case C such that the average strain rate in the structure remains close to 1 %/min and thus consistent with material characterization. The measured load was found to be linearly proportional and this proportionality constant gives us the effective stiffness of the structure. Given the planar nature of the deformation, we employed digital image correlation (DIC) to obtain the full displacement field from the experiments. The results are shown in Fig. S21 (bottom row) along with the simulation results. The range of displacements from both experiments and simulations agree well.

However, we found the deformation in the *y*-direction adjacent to the hole to be localized in the simulations but not in the experiments. A high-fidelity simulation of the structure should reveal the source of discrepancy but is beyond the scope of this paper.

The laminated plate structure was simply loaded by placing the printed structure with an elevated supporting frame on a flat plate. A pneumatic gripper attached to the crosshead was used to grip and push the vertical tab downwards at a rate of 0.4 mm/min which equates to an average strain rate of 1%/min in the structure. We used a 100 N load cell given the lower loads needed to bend the plate (~1 N).

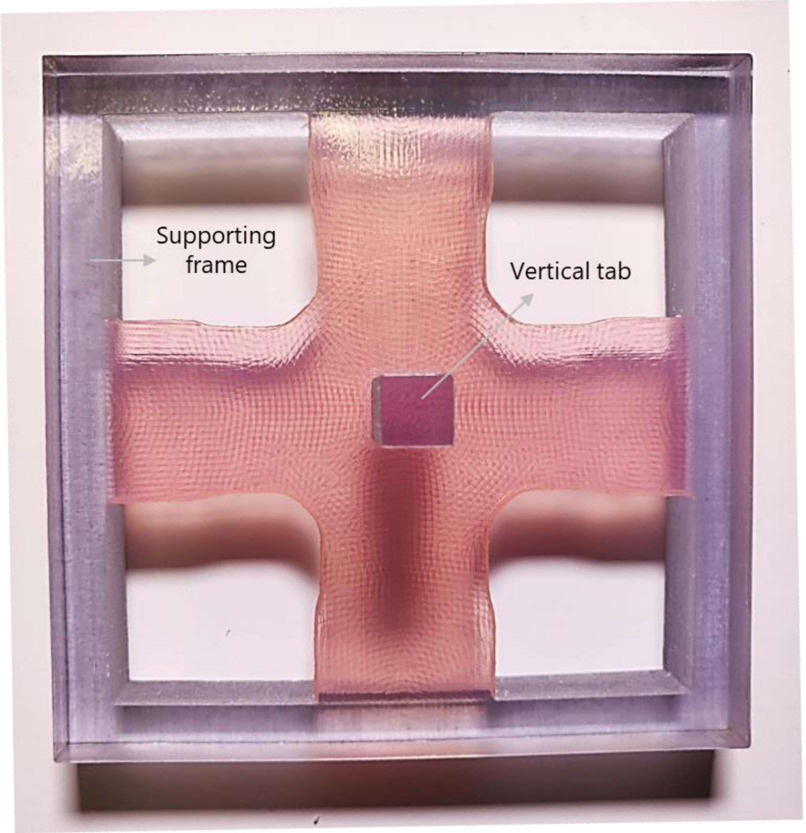

Figure S20 Laminated plate – 3D printed optimized structure with a supporting frame and a vertical tab to facilitate clamping of the outer boundaries and bending load on the structure respectively

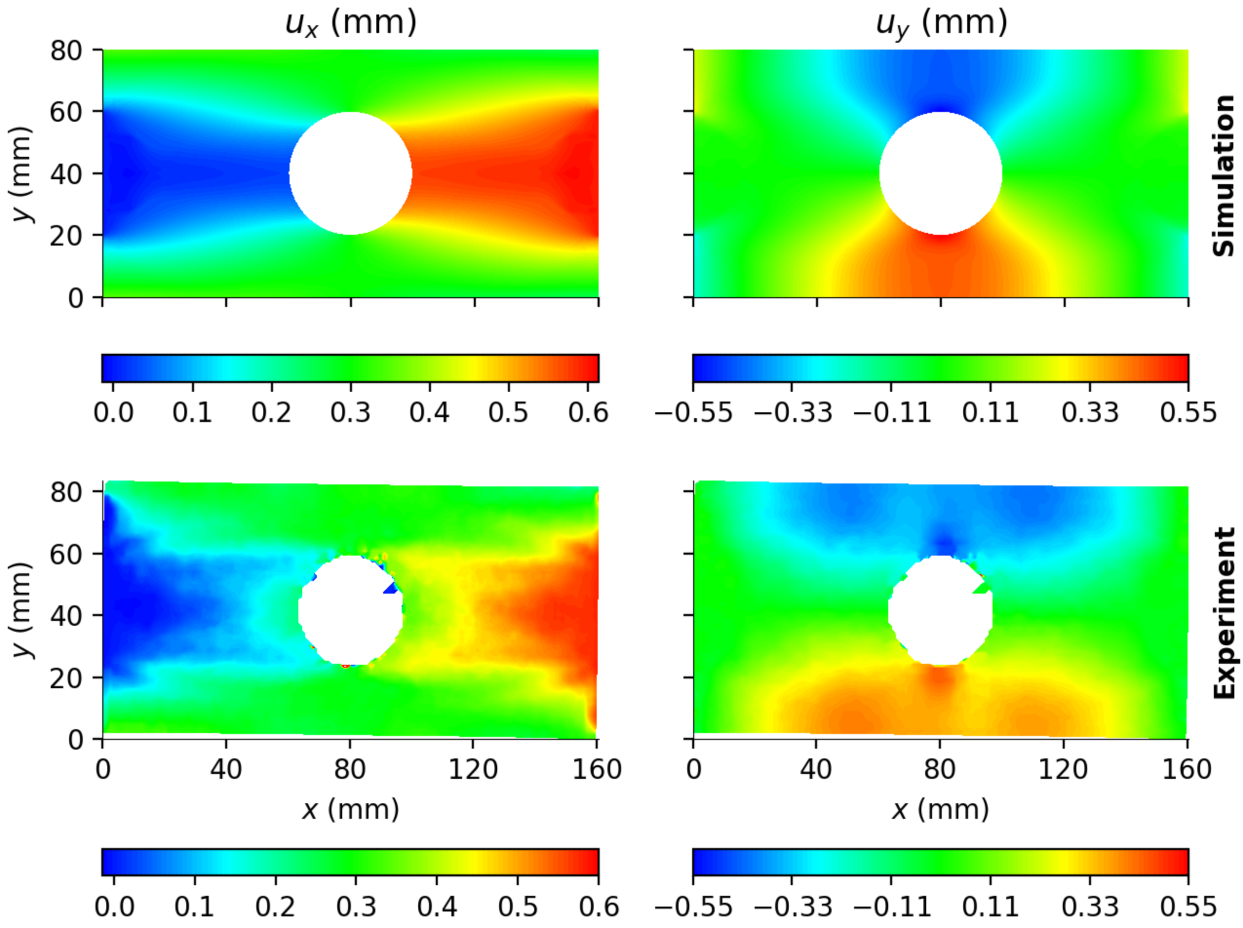

Figure S21 Plate with a hole (case B) – comparison of displacements obtained with digital image correlation and simulations

3.3 Finite Element Analysis with Defects

We set up finite element analysis, while still using the Mori-Tanaka homogenization method, to understand the effect of the singularities/defects generated during the material compilation step. We chose the plate with a hole problem owing to its planar nature and thus low computational complexity. The defects were identified as discontinuities in the fibers as shown in Fig. S15 and located approximately by the open fiber ends (fibers that are too short and fiber ends on the boundaries are ignored). We introduced these defects into the FE model by simply setting the local material volume fraction to zero within a circular region surrounding the open fiber ends while using the optimized fiber volume fractions elsewhere as shown in Fig. S22. The results from these simulations for the three cases A, B, and C are tabulated in Table S3. These FE simulations with defects qualitatively capture the effect of the defects and suggest that the deviation from the ideal performance increases with defect density, thus validating the assumption we made in the main text. When compared to the experiments, we get a good agreement for case B. The reason for the disagreement in case A could be due to insufficient scale separation whereas the disagreement in case C could be attributed to undesired deformations in the printed structure due to excessive swelling of Tango+ matrix material.

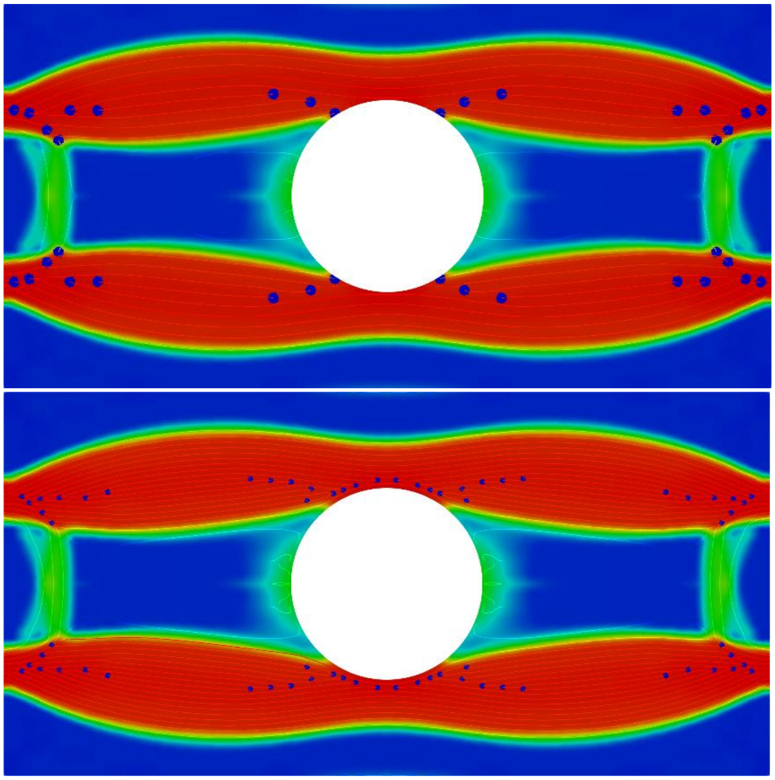

Figure S22 Plate with a hole – finite element model with defects introduced for case A (top) and case B (bottom)

Table S3 Comparison of experiments and simulations with and without defects introduced

Dimensions (cm×cm×cm)	*Number of Defects*	*Defect Density (cm^{-3})*	*Effective Stiffness (N/mm)*		
			Simulation (no defects)	*Simulation (with defects)*	*Experiment*
8 × 4 × 0.8 (1X)	292	12.65	84.36	64.28	50.55
16 × 8 × 0.8 (2X)	692	7.49		70.816	69.10
24 × 12 × 0.8 (3X)	952	4.58		76.57	64.58

4. Digital Manufacturing via Automated Fiber Placement

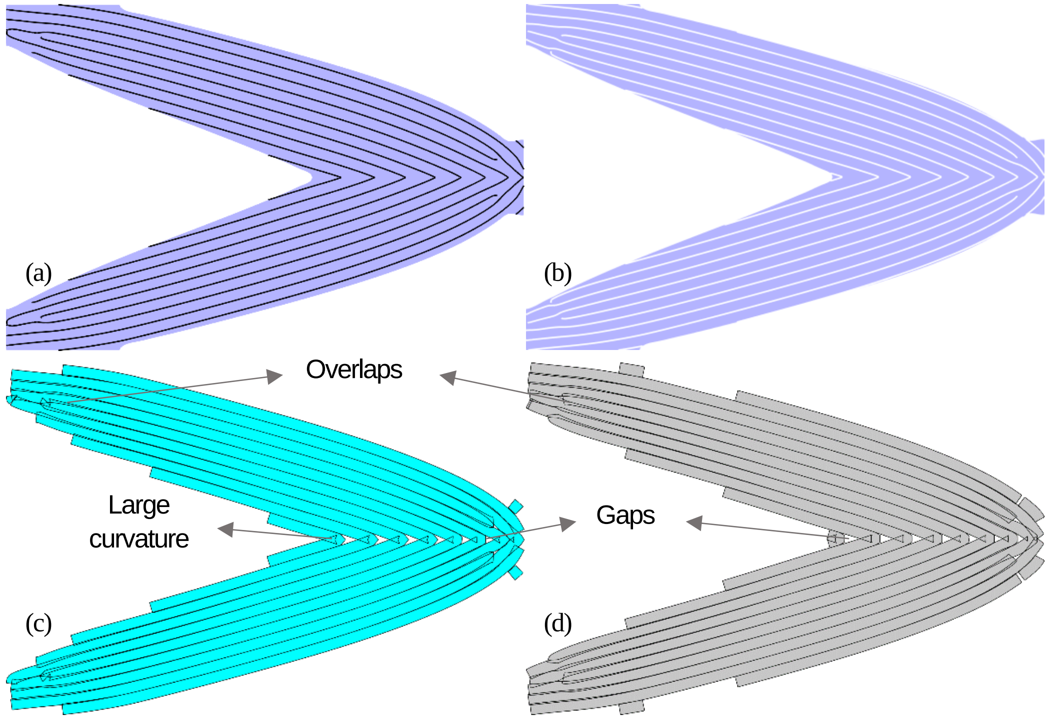

Figure S23 Optimized 2D short beam (120×80 cm): (a, b) show the fiber layout (black in (a) and white in (b)) with a spacing of 1 in. on top of the optimized macroscale structure. Fiber layout in (b) is offset by half of the fiber spacing. (c, d) show illustrations of tape layup with ribbons centered about the fiber trajectories in (a) and (b) respectively.

Figure S23 illustrates how automated fiber placement (AFP) can, in principle, be adapted for the digital manufacturing step of our workflow. We use the stiff 2D short beam example from Fig. 1 of the main text here. We scaled the structural dimensions by 10 times to 120×80 cm and picked the fiber spacing to be 1 in., a standard dimension for fiber tapes used in AFP and similar processes. Figures S23(a) and (b) show the optimized fiber layouts obtained from the material compilation step with the difference being curves in (b) are offset by 0.5 in. The curves in these layouts can serve as trajectories along which fiber tape can be laid down. Figures S23(c) and (d) show *ribbons* of width 1 in. obtained by extruding the individual curves along the in-plane normal direction. The ribbons then represent the laid down tapes and it can be seen that we get excellent coverage. The figures also highlight gaps and overlaps occurring near singularities and regions of high curvature. Alternating (a) and (b) layouts during layup will help cover the gaps. High curvature regions could be handled via tow drops. Alternatively, a constraint on maximum curvature could be imposed. This could be achieved by coupling the design automation and material compilation steps and imposing a global constraint on the total curvature.

5. References

1. Sigmund, O. & Maute, K. Topology optimization approaches: A comparative review. *Structural and Multidisciplinary Optimization* vol. 48 1031–1055 (2013).
2. Svanberg, K. A Class of Globally Convergent Optimization Methods Based on Conservative Convex Separable Approximations. *SIAM J. Optim.* **12**, 555–573 (2002).
3. Boddeti, N., Rosen, D. W., Maute, K. & Dunn, M. L. Multiscale optimal design and fabrication of

laminated composites. *Compos. Struct.* **228**, 111366 (2019).

4. Guest, J. K., Prévost, J. H. & Belytschko, T. Achieving minimum length scale in topology optimization using nodal design variables and projection functions. *Int. J. Numer. Methods Eng.* **61**, 238–254 (2004).

5. Wang, F., Lazarov, B. S. & Sigmund, O. On projection methods, convergence and robust formulations in topology optimization. *Struct. Multidiscip. Optim.* **43**, 767–784 (2011).

6. Schwarze, M. & Reese, S. A reduced integration solid-shell finite element based on the EAS and the ANS concept-Geometrically linear problems. *Int. J. Numer. Methods Eng.* **80**, 1322–1355 (2009).

7. Knöppel, F., Crane, K., Pinkall, U. & Schröder, P. Stripe patterns on surfaces. *ACM Trans. Graph.* **34**, 1–11 (2015).

8. Boddeti, N., Ding, Z., Kaijima, S., Maute, K. & Dunn, M. L. Simultaneous Digital Design and Additive Manufacture of Structures and Materials. *Sci. Rep.* **8**, 15560 (2018).

9. Ding, Z. *et al.* Direct 4D printing via active composite materials. *Sci. Adv.* **3**, e1602890 (2017).

10. Ding, Z., Weeger, O., Qi, H. J. & Dunn, M. L. 4D rods: 3D structures via programmable 1D composite rods. *Mater. Des.* **137**, 256–265 (2018).
